# Optimizing a carbon source based on water temperature to reduce *Vibrio* abundance and promote beneficial bacteria in a *Penaeus vannamei* shrimp biofloc system in China: a preliminary study

**DOI:** 10.3389/fsysb.2026.1819518

**Published:** 2026-06-22

**Authors:** Abdallah Ghonimy, Zhao Chen, Laura Susana López Greco, Fernando Dyzenchauz, Jian Li

**Affiliations:** 1 Key Laboratory of Sustainable Development of Marine Fisheries, Yellow Sea Fisheries Research Institute, Chinese Academy of Fishery Sciences, Qingdao, China; 2 Laboratory for Marine Fisheries Science and Food Production Processes, Qingdao National Laboratory for Marine Science and Technology, Qingdao, China; 3 Laboratorio de Biología de la Reproducción y el Crecimiento de Crustáceos Decápodos, Departamento de Biodiversidad y Biología Experimental, Facultad de Ciencias Exactas y Naturales, CONICET, Instituto de Biodiversidad y Biología Experimental y Aplicada (IBBEA), Universidad de Buenos Aires, Buenos Aires, Argentina

**Keywords:** biofloc, metabolites, microflora, shrimp, temperature

## Abstract

Biofloc technology (BFT) does not recognize bioactive molecules, which significantly impact shrimp intestinal microbiota and *Vibrio* bacterial abundance, and this is a critical industrial issue. This study investigated the effects of temperature (22 °C during onset of the culture season and 28 °C during the culture season) and carbon sources (starch, seaweed, and their mix) on *Penaeus vannamei* in a 60-day trial. Liquid chromatography–mass spectrometry (LC–MS/MS) analysis revealed high L-carnitine (450-fold) and mesaconic acid (12-fold) in Mix-28, correlating with increased Proteobacteria and reduced *Vibrio* abundance. Irisflorentin and probucol, bacterial virulence antagonists, were also elevated. Starch-22 exhibited lower *Vibrio* levels, while seaweed treatments showed higher telmisartan (three-fold) at both temperatures. Mix-22 had elevated isobutyric acid (six-fold). Our findings indicate that carbon complexity influences microbial composition, with starch (22 °C) and mix (28 °C) recommended for pre-season (during onset of the culture season) and season BFT cultures, respectively. The bioactive molecules telmisartan, bile acids, mesaconic acid, irisflorentin, and probucol are proposed as shrimp prebiotics.

## 1 Introduction

The world’s population growth is increasing food demand and necessitates aquaculture production (Bohnes and Laurent, 2021). In 2022, global aquaculture production surged to 130.9 million tons, representing 58% of global aquaculture and fisheries production, of which aquatic animals represented 72.6% and algae 27.4% (FAO, 2022). From a circular economy perspective, integrating algae to enhance biofloc aquaculture system biosecurity represents a promising and sustainable approach to improving aquaculture production. Intensive aquaculture production comes with challenges of decreased water quality and increased disease outbreaks in the culture systems (Aaronson and Horvath, 2002; Chang et al., 2020; Mingming et al., 2020). Such management difficulties are commonly manipulated by water exchange (Bohnes and Laurent, 2019; Ottinger et al., 2016).

Shrimp are one of the most widely cultured aquaculture species in biofloc systems due to their ability to utilize biofloc as a feed source, which affects their intestinal microbial composition (Ghonimy et al., 2023a). Biofloc technology (BFT) and clear-water recirculating aquaculture systems are common closed systems in the aquaculture industry (Ray et al., 2017). In BFT, carbohydrate supplements added to the culture water enable heterotrophic bacterial activity, which can rapidly remove toxic ammonia through direct bacterial assimilation from the water (Brandão et al., 2021; De Schryver and Verstraete, 2009) and generate suspended solids containing uneaten feed particles, fecal matter, detritus, and a variety of microorganisms, including bacteria, algae, and zooplankton (Burford et al., 2004; Moss and Pruder, 1995; Ray et al., 2010).

Nutrient availability forms the microbial structure by simulating the relevant physiological functions (Brandão et al., 2021; Wang et al., 2021; Xavier et al., 2022). Organic carbon is an important nutrient for heterotrophic bacterial activity, where the quantity and timing of supplementation can manipulate shrimp’s intestinal bacterial composition (Ghonimy et al., 2023b). Other studies have demonstrated the effects of different plant-derived materials and algae on disease control and their underlying mechanisms mediated by bioactive compounds (Khanjani et al., 2024). Seaweed-related industrial activities generate large quantities of seaweed byproduct in China, Korea, and Japan (Abakari et al., 2021) since traditional alginate production discards approximately 80% of raw materials (Wekre et al., 2023). Seaweed has an antibacterial activity against pathogenic bacteria due to its content of polyphenol compounds (Xu et al., 2018); because of that, seaweed is strongly nominated as a sustainable carbon source for BFT, while the massive production of seaweed byproduct, not competing with human food, reveals its potency as an alternative carbon source for BFT. A recent study demonstrated that adding molasses as a carbon source to a shrimp biofloc system reduced the abundance of *Vibrio* spp. (Gustilatov et al., 2026); however, starch was selected as a chemically defined, readily degradable carbon source with consistent quality in contrast to more complex commercial sources such as molasses. Maize starch, a globally dominant source, has been selected for its superior cold-water solubility compared to other common starches such as sweet potato and tapioca (Chenlu et al., 2021). This choice has provided a clear benchmark for carbon availability, against which the performance of seaweed—a structurally complex substrate with a slower carbon release profile and associated bioactive compounds—could be evaluated (Miao et al., 2020; Tinh et al., 2021). In BFT, water temperature mediates floc formation by inducing heterotrophic bacterial activity (De Schryver et al., 2008; Dunstall and Schwarz, 1983). Certainly, low water temperature decreases the BFT flocculation rate due to its effect on heterotrophic bacterial activity—the main bacterial group responsible for flocculation (Wilén et al., 2000). As an example, total suspended solids concentration was approximately 990 mL g^−1^ at 33 °C and 750 mL g^−1^ at 21 °C (Wilén et al., 2000). Microbial flocs disturb the quorum sensing (signal molecules regulate the bacterial gene expressions based on bacterial community density) of pathogenic bacteria in BFT-raised brine shrimp *(Artemia franciscana*) (Crab et al., 2010; El-Sayed, 2021) and reduce *Vibrio* abundance in shrimp (*Litopenaeus vannamei*) BFT (Ghonimy et al., 2023a). Water temperature affects the TSS level (Hostins et al., 2015), and because TSS contains different bacteria and organic molecules (Burford et al., 2004; Moss and Pruder, 1995; Ray et al., 2010), temperature is expected to affect the organic composition of TSS. In our previous study (Ghonimy et al., 2023b), biofloc chemical composition modulated shrimp intestinal microbial diversity. In this context, carbon source and temperature may play an integrated role in increasing the biosecurity status of BFT.

The intestinal microbiota are commonly considered an additional organ for the host since their effects extend beyond intestinal digestive efficiency by influencing the host’s immunity and health (Ai et al., 2018; Ge et al., 2018). In shrimp, their intestinal microbiota can be modulated by the microbiota of the culture water (Deng et al., 2019; Giatsis et al., 2015). This water–intestinal microbiota interaction is the most complex relationship between environment and host in BFT compared to other intensive aquaculture systems, since high microbial density and the bacterial accumulated bioactive molecules could directly affect shrimp intestinal microbiota composition through their ingestion of biofloc (Abakari et al., 2022; Montalban-Arques et al., 2015). Viable microbes and dead bacterial cells, along with their metabolic content, may be regarded as important elements in the environment–host microbial interaction through ingestion by shrimp (Tepaamorndech et al., 2020). For example, BFT water showing a high *Vibrio* abundance also shows increased abundance in the intestine of Pacific white shrimp (Tepaamorndech et al., 2020).

The intense bacterial density in BFT likely generates a high level of bacterial metabolic content in the form of “bioactive molecules.” These molecules reach the shrimp intestine through the direct ingestion of biofloc containing bacteria and their bioactive molecules. This could play a key role in shaping the intestinal microbiota structure. However, little is known of the underlying mechanism of the interaction between shrimp intestinal microbial composition and biofloc content (Ai et al., 2018). Biofloc composition is affected by bacterial activities, which are influenced by water temperature and nutrient availability. Mechanistic data from the untargeted molecule analysis of bioflocs could help reveal the underlying mechanism of the interaction between BFT environment and shrimp intestinal microbiota composition. To the best of our knowledge, this is the first experiment to explore the effect of water temperature on metabolite content in BFT and intestinal bacterial composition in *Penaeus vannamei*. We hypothesize that water temperature could affect BFT metabolite content and, subsequently, shrimp intestinal microbial composition by mediating the effect of carbon source on BFT. This study was designed to investigate the effect of temperature and carbon sources on the bioactive molecule composition of bioflocs and the microbiota composition of shrimp. A simple carbon source could be suitable for a low-temperature condition, encouraging heterotrophic bacterial activity, while a certain complexity of a carbon source will favor the growth of heterotrophic bacteria over *Vibrio* bacteria, since carbon release over a longer period will provide continuous carbon availability for heterotrophic bacterial dominance. Furthermore, bioactive secondary metabolites derived from seaweed, a complex carbon substrate, can directly suppress *Vibrio* proliferation. This inhibition alters the competitive landscape, favoring the growth of heterotrophic bacteria by reducing competition for available carbon resources.

## 2 Materials and methods

### 2.1 Experimental design

**FIGURE 1:**
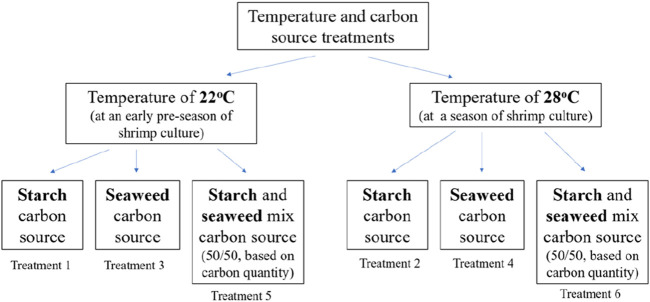
Experimental design for *Penaeus vannamei* exposition during 2-month growth in biofloc (BFT) with different carbohydrate sources and temperatures. “Pre-season temperature” refers to the time of late autumn and early summer while preparing a new BFT for the warm culture season, while “season temperature” refers to the time of warm summer during the breeding season. Starch-22 (T1), starch carbon source at 22 °C; Starch-28 (T2), starch carbon source at 28 °C; Seaweed-22 (T3), seaweed carbon source at 22 °C; Seaweed-28 (T4); seaweed carbon source at 28 °C; T5 (Mix-22), starch–seaweed mix carbon sources at 22 °C; T6 (Mix-28), starch–seaweed mix carbon sources at 28 °C.

This study applied different carbon sources (starch, seaweed, and a 50/50 starch–seaweed mix) based on carbon content with different temperatures (22 °C and 28 °C). These temperatures correspond to the average rearing temperatures for *P. vannamei* in the autumn and summer seasons, respectively, in Shandong Province, China. The optimal rearing temperature for *P. vannamei* shrimp is 28 °C (Mai et al., 2025; Prates et al., 2023). The six treatments were conducted in triplicate (Figure 1). Cylinder-shaped PVC aquaria (450 L) were used as BFT experimental units (filled up to 250 L) with a zero-water exchange rate. The shrimp were provided by Hainan Zhengtai No. 1 Aquatic Seed Co., Ltd. (Wenchang, Hainan Province, China). They were fed at a rate of 5% of their total biomass unit on daily basis, three times per day (8:00 a.m., 12:00 a.m., and 4:00 p.m.) (Adineh et al., 2023; Long et al., 2023) for the 28 °C treatments, while the same feeding practices were applied to the 22 °C treatments with a lower feeding rate of 1.7% of their total biomass unit. The feeding rates were initially calculated based on the apparent feed intake (the ability to consume a certain amount of feed in 1 day) for equal shrimp densities under the differing temperatures of 22 °C and 28 °C, and then the shrimp density was adjusted for the 22 °C treatments based on equal feed intake for each experimental unit. The calculation was based on the principle of compensating for temperature-dependent differences in individual shrimp feed intake. The formula applied was as follows:

$$\text{Stocking Density }\left(\text{per tank}\right)=\left(\text{Target Daily Nitrogen Input}\right)/\left(\text{Individual Daily Feed Intake at}\text{ given Temperature}\times \text{Feed Nitrogen Content}\right).$$

A total of 185 juvenile *L. vannamei* shrimp (1.6 ± 0.3 g, 5.7 ± 0.4 cm) were allocated for each experimental unit of 22 °C treatments, whereas 65 juvenile shrimp (1.6 ± 0.3 g, 5.7 ± 0.4 cm) were allocated to the 28 °C treatments. Shrimp density was adjusted to ensure equal nitrogen input across experimental treatments since shrimp’s feeding consumption (Hostins et al., 2015) and growth rate are not equal at different culture temperatures of 20, 24, and 28 °C in BFT systems (Prates et al., 2023). This study focuses on examining how various carbon sources and their mixtures influence nitrogen assimilation in BFT systems. Supplying equal amounts of nitrogen to BFT systems across different temperatures allows unbiased comparisons among treatments by eliminating variations in feed consumption—the main nitrogen source—caused by temperature differences. The function of BFT systems is mainly governed by available carbon and nitrogen in the culture medium. Nitrogen input is largely determined by shrimp feed intake, which fluctuates with water temperature. At the optimal temperature of 28 °C, shrimp maintain normal feed consumption, delivering consistent nitrogen levels to the biofloc. At the suboptimal temperature of 22 °C, shrimp show lower feed intake due to reduced metabolism, slower enzyme activity, and reduced activity, leading to less nitrogen release into the biofloc. Thus, to ensure valid comparisons among BFT treatments, equal nitrogen input across all units was essential. Since shrimp are not the primary focus of this study, shrimp density was adjusted at different temperatures to standardize total feed and nitrogen supply across all experimental BFT units. The Animal Ethics Committee of the Yellow Sea Fisheries Research Institute (YSFRI), Chinese Academy of Fishery Sciences (CAFS), determined and approved that our methodology complied with the national ethical and animal welfare standards regarding the use of animals for research.

A commercial diet with a crude protein content of 41% and crude fat content of 4% was used; in addition, crude fiber, ash, lysine, and total phosphorus at 6%, 15%, 2%, 0.8%, and 12%, respectively (Alpha Feed®, Huai’an, Jiangsu, China) were applied to the experimental shrimp for 2 months. The carbon source was added at a ratio (C/N = 15:1) based on dietary nitrogen content after 30 min of first feeding (8:30 a.m.) daily. The C/N ratio of the carbon source for feed nitrogen content was set at 15 based on our previous research on BFT shrimp systems (Ghonimy et al., 2023a). Seaweed *Laminaria* by-product powder (particle diameter 0.85 mm) of alginate extraction was used as a carbon source. The chemical composition of seaweed byproduct and maize starch is presented in Table 1. Two different carbon sources in three combinations were tested: starch, seaweed by-product, and starch mixed with the seaweed by-product. These three combinations were applied at 22 °C and 28 °C.

**TABLE 1:** Chemical composition of carbon sources.

Nutrient (% ± SE)	Seaweed by-product	Maize starch
Crude protein	17 ± 1.5	1.0 ± 0.2
Crude fat	21 ± 2.0	0.1 ± 0.05
Carbohydrate	61 ± 3.0	86 ± 2.5
Ash	4.0 ± 0.5	0.1 ± 0.02
Moisture	7.0 ± 0.8	12 ± 1.0
NFE	30 ± 3.5	0.8 ± 0.1
Carbon content	54 ± 4.0	38 ± 3.0

The respective amounts of seaweed and starch added were 9.1 g and 12.8 g for the 22 °C treatments and 9.4 g and 13.3 g for the 28 °C treatments, where the mix treatment received the half of each carbon source under the 22 °C and 28 °C conditions. These carbon source amounts were calculated to match the C/N ratio of 15:1 based on each carbon source’s carbon content, with carbon content of 53% in protein, 86% in fat, and 44% in carbohydrate (Rouwenhorst et al., 1991), and nitrogen content of 16% in protein (Rouwenhorst et al., 1991). The seaweed cell-wall structure traps nitrogenous compounds, but the microbial enzymes dissociate part of these protein compounds (Maiorano et al., 2022). In the present study, precipitated seaweed particles were observed in significant quantities during the experimental period; the seaweed proteins were mostly trapped, while the free functional proteins did not significantly participate in the nitrogen pool for bacterial activity. During the experiment, the evaporated water was compensated for by the daily addition of dechlorinated fresh water to maintain a constant water column in the culture aquaria to avoid the effects of evaporation on salinity changes. High-throughput sequencing analysis and liquid chromatography linked to tandem mass spectrometry (LC–MS/MS) (1290 Infinity LC, Agilent Technologies, United States) were performed for the filtered biofloc samples (one sample per replicate aquarium) and shrimp intestinal bacterial community (three shrimp intestines per replicate aquarium), respectively, after an experimental period of 2 months. At the beginning of the BFT experiment, biofloc volume was measured for all treatment units by an Imhoff cone (0.5 ± 0.0 mL), followed by another biofloc volume measurement for all treatment units by the end of the 60-day experiment. Biofloc maturation was determined based on the ability of a biofloc system to maintain nitrate and nitrite levels at the accepted level for shrimp culture (Llario et al., 2019).

The growth performance indicators were calculated using standard aquaculture formulas. The specific growth rate (SGR, %/day) was determined as (Ln (final body weight) − Ln (initial body weight))/culture period (days) × 100. The feed conversion ratio (FCR) was calculated as total dry diet fed (kg)/total wet weight gain (kg). The protein efficiency ratio (PER) was derived from final wet weight gain (g)/protein intake (g).

### 2.2 Water quality parameters

Water salinity, pH, temperature, and dissolved oxygen were measured using a YSI Incorporated device (Yellow Springs, OH, United States), while NH^4+^, NO_3_^−^, and NO_2_^−^ were determined using a QuAAtro Nutrient AutoAnalyzer (SEAL Analytical Ltd., Germany).

### 2.3 Extraction of metabolites

Liquid chromatography coupled with tandem mass spectrometry (LC–MS/MS) was used for non-targeted metabolite analysis as per Dunn et al. (2013), with data processing according to a zero level of confidence in addition to the standard four levels from 1 to 4 (Blaženović et al., 2018). The supernatant of each water sample (50 mL) was collected after filtration by 0.22 µm-filter paper and mixed with a pre-cold methanol/acetonitrile/aqueous solution (2:2:2, v/v, with volume of 400 μL); the sample was then vortexed using a vortex instrument (Haimen City Qilin Medical Instrument Factory, Haimen, Jiangsu, China) for 60 s. Subsequently, it was centrifuged using a centrifuge instrument (Xiangyi Centrifuge Instrument Co., Ltd., Changsha, Hunan, China) at 14,000 g at −20 °C for 20 min. Then, the supernatant was vacuum-dried using a vacuum concentrator (Eppendorf LTD., Hamburg, Germany) and placed in 100 μL aqueous solution of acetonitrile (acetonitrile: water = 1:1, v/v), and then the sample was vortexed for 60 s followed by centrifugation at 14,000 *g* at 4 °C for 15 min. The resultant mixture was used for LC–MS/MS analysis after filtration through a 0.22 µm syringe filter. All samples were handled in three biological replicates.

### 2.4 LC–MS/MS condition

Metabolite separation was performed as per Dunn et al. (2013): the samples were processed by UHPLC (infinity LC ultra-high-performance liquid chromatography) using an Agilent 1290 instrument (Agilent Technologies, Santa Clara, CA, United States) coupled with a quadrupole time-of-flight (AB Sciex TripleTOF 6600) and connected to an HILIC column for the separation process (1.7 µm, 100 × 2.1 mm, Waters ACQUITY UPLC BEH Amide). The TOF MS scan m/z range was 60–1000 Da, the product ion scan m/z range was 25–1,000 Da, the TOF MS scan accumulation time was set at 0.20 s/spectra, the time accumulation of the product ion scan was 0.05 s/spectra, the declustering potential (UU dp) was set at ± 60 V (±2 modes), and collision energy was presented as 35 ± 15 eV. IDA was set as follows: candidate ions were 10 per cycle, and isotopes within 4 Da were excluded (Wang et al., 2022). The compound identification of metabolites was performed by comparing the accurate m/z value (<10 ppm) and MS/MS spectra with an in-house database established using available authentic standards. The total ion chromatogram (TIC) of the quality control (QC) sample was compared with the overlapping spectra. The experimental results showed that the response intensity and retention time of each chromatographic peak largely overlapped, indicating that the variation caused by instrumental error is minimal throughout the experimental process.

### 2.5 Bacterial intestinal community analyzed by high-throughput sequencing analysis

Three full shrimp intestines from each sample replicate were used to analyze the total DNA after a 60-day experimental period. They were extracted using a TIANamp Bacteria DNA Kit (Tiangen Biotech, Beijing, China), and agarose gel electrophoresis check confirmed DNA integrity. DNA concentration was measured using a NanoDrop Spectrophotometer (Thermo Scientific, United States). The V3–V4 region of the 16S rRNA gene with the primers 806R (5′-GGACTACHVGGGTWTCTAAT-3′) and 338F (5′-ACT​CCT​ACG​GGA​GGC​AGC​AG-3) (Xu et al., 2016) was amplified by polymerase chain reaction (PCR) using a MyCycler^TM^ thermal cycler (BIO-RAD, United States). The DNA was purified and then sequenced using Illumina MiSeq. In order to ensure the accuracy and reliability of subsequent data analysis, a low cycle number of amplification and uniform amplification cycles were used for each sample, where randomly selected samples for the preliminary experiments were prepared for amplification at the lowest number of cycles with appropriate concentrations of products for all samples. The stability of reference genes was assessed using geNorm; GAPDH and β-actin showed M-values of 0.42 and 0.48, respectively, confirming their suitability. After the pre-experiment was completed, the PCR was officially tested using ProTaq. The primers used in this qPCR assay underwent a multi-tiered validation process to guarantee specificity. Initially, primers were designed and evaluated *in silico* against a comprehensive 16S rRNA sequence database (SILVA) to predict coverage and theoretical specificity. Empirical validation was then performed to confirm actual performance. The specificity of each primer pair was tested *via* qPCR using control templates and was confirmed by the presence of a single, sharp peak in post-amplification melting curve analysis, indicating amplification of a single, specific product. Furthermore, amplification products were visualized by agarose gel electrophoresis to verify a single amplicon of the expected size. The quantitative reliability of the qPCR assay was established by generating standard curves using serial dilutions of target DNA; only assays demonstrating a robust linear relationship, with a coefficient of determination (*R* ^2^) typically >0.99, were used for final quantification.

Alpha diversity, reflecting the richness and evenness of microbial communities within each sample, was assessed using three standard indices calculated from the 16S rRNA gene amplicon sequencing data. To account for undetected rare species and estimate total species richness, the bias-corrected Chao1 estimator was applied. Community diversity, which incorporates both species richness and their relative abundances, was quantified using the Shannon–Wiener index. The Simpson index (1-D), an index less sensitive to rare species, was also calculated to measure the probability that two randomly selected reads belong to different species. All indices were computed using the Vegan package (version 2.6-4) in R.

### 2.6 Statistical analysis

UHPLC data normalization and processing were performed using the R package (ropls), and multivariate data analysis was applied, including orthogonal partial least-squares discriminant analysis (OPLS-DA) and Pareto-scaled principal component analysis (PCA). The robustness of the model was tested using seven-fold cross-validation and response permutation tests. To indicate the variable importance in the projection (VIP) value contribution to the resulting classification, the OPLS-DA model was used. First, preliminary screening was performed based on VIP values from the OPLS-DA model. Subsequently, to enhance screening rigor and identify potentially important metabolites, Student’s t-tests were conducted on all metabolites (including those with VIP < 1). Finally, metabolites meeting either of the following criteria were defined as differential metabolites for subsequent analysis: (1) VIP value > 1; (2) VIP value < 1 but t-test *p*-value < 0.05. PCA is a multivariate data analysis method used to investigate intergroup classification trends and identify data outliers. OPLS-DA is another data classification method that separates the independent and dependent variables for clearer interpretation of intergroup variation (Chen et al., 2022; Kalu et al., 2021).

FLASH software was used to splice the high-throughput sequencing paired-end (PE) reads (Magoč and Salzberg, 2011) according to Fastp and the overlap relationship (Chen et al., 2018) for sequencing quality control. UPARSE (Edgar, 2013) software was used for operational taxonomic unit (OTU) clustering and biological information statistical analysis for the sequence with a similarity of 97% (Edgar, 2013; Stackebrandt and Goebel, 1994). RDP Classifier (Wang et al., 2007) software was used for taxonomic classification of each sequence. Alpha diversity was calculated using MOTHUR (Schloss et al., 2009). SPSS Statistics 22 was used to perform statistical analysis to determine data differences, with a *p-*value < 0.05 considered significant (Liu et al., 2018).

## 3 Results

This study investigated the effects of temperature (22 °C and 28  C) and carbon source (starch, seaweed, and their mix) on the profile of biofloc bioactive molecules and the intestinal microbiota composition of *Penaeus vannamei*. The resultant data were categorized into the following groups: common indicator data (biofloc volume, body weight, survival rate, and water quality), biofloc bioactive molecules profile (bacterial activator factors, bacterial inhibition factors, xenobiotic factors), and shrimp intestinal microbiota composition (bacterial community indices, bacterial composition at phylum, genus, and species levels).

### 3.1 Biofloc volume, growth performance, survival rate, and water chemical parameters

The biofloc volume measurement showed significant differences among different treatments, with the 28 °C treatments showing higher biofloc volumes than at 22 °C (*p* < 0.05) (Table 2). Across both experimental temperature regimes, starch as a sole carbon source yielded the highest biofloc volume. This was followed by the starch–seaweed mixture, with seaweed alone producing the lowest volume among the tested carbon substrates. The survival rate and final body weight of the shrimp were not significantly different between the 22 °C and 28 °C treatments (*p* > 0.05) (Table 1). The growth performance parameters for the 60-day trial showed clear differences between temperature treatments. The specific growth rate (SGR) was 1.21%/day at 22 °C and 2.41%/day at 28 °C. The feed conversion ratio (FCR), calculated from feeding rates of 1.7% and 5.0% of biomass, was 1.48 at 22 °C and 2.46 at 28 °C. The protein efficiency ratio (PER), based on a 41% crude protein diet, was 1.64 at 22 °C and 0.99 at 28 °C. These results indicate that the optimal 28 °C temperature promoted a faster growth rate, while the suboptimal 22 °C temperature, under a restricted feeding regime, yielded better feed and protein conversion efficiency. The monitored water quality parameters showed values within the normal range for shrimp culture (Xu et al., 2016), where salinity was 28 ± 1 g L^−1^, dissolved oxygen was 5.9 ± 0.8 mgL^−1^, NH_4_^+^ was 0.018 ± 0.004 mgL^−1^, NO_2_^−^ was 0.06 ± 0.003 mgL−1, NO_3_^−^ was 0.54 ± 0.04 mgL^−1^, and pH was 7.6 ± 0.3 (Table 2).

**TABLE 2:** Biofloc volume, growth performance, and survival rate of *Penaeus vannamei* after a 2-month growing period in biofloc (BFT) with different carbohydrate sources and temperatures.

Treatment^a^	Biofloc volume (mL)	Growth performance				Survival rate (% ± SE)	Water chemical parameter (initial vs. final)					
		Final body weight (g ± SE)	Specific growth rate (SGR, %/day ± SE)	Feed conversion ratio (FCR)	Protein efficiency ratio (per)							
							Salinity (g L^−1^ ± SE)	Dissolved oxygen (mgL^−1^ ± SE)	NH_4_^+^ (mgL^−1^ ± SE)	NO_2_^−^ (mgL^−1^ ± SE)	NO_3_^−^ (mgL^−1^ ± SE)	pH (± SE)
Starch-22	12 ± 3^c^	3.5 ± 0.6^b^	1.21 ± 0.01^b^	1.48 ± 0.01^b^	1.64 ± 0.01^b^	93 ± 1.5^a^	27 ± 1^a^ 28 ± 1^a^	7.9 ± 0.3^a^ 6.2 ± 0.2^a^	0.17 ± 0.006^a^ 0.019 ± 0.002^a^	0.13 ± 0.001^a^ 0.024 ± 0.002^a^	0.46 ± 0.001^a^ 0.5 ± 0.01^a^	8.56 ± 0.01^a^ 7.81 ± 0.01^a^
Starch-28	20 ± 5^a^	6.5 ± 0.3^a^	2.41 ± 0.03^a^	2.46 ± 0.03^a^	0.99 ± 0.03^b^	89 ± 3^b^	27 ± 1^a^ 28 ± 1^a^	7.9 ± 0.3^a^ 5.1 ± 0.3^b^	0.17 ± 0.006^a^ 0.015 ± 0.003^a^	0.13 ± 0.001^a^ 0.022 ± 0.001^a^	0.46 ± 0.001^a^ 0.58 ± 0.02^a^	8.56 ± 0.01^a^ 7.4 ± 0.02^b^
Seaweed-22	8.0 ± 2^d^	3.27 ± 0.5^b^	1.21 ± 0.01^b^	1.48 ± 0.01^b^	1.64 ± 0.01^b^	92 ± 1.8^a^	27 ± 1^a^ 28 ± 1^a^	7.9 ± 0.3^a^ 6.6 ± 0.3^a^	0.17 ± 0.006^a^ 0.022 ± 0.002^a^	0.13 ± 0.001^a^ 0.014 ± 0.001^b^	0.46 ± 0.001^a^ 0.52 ± 0.01^b^	8.56 ± 0.01^a^ 7.9 ± 0.02^a^
Seaweed-28	15 ± 3^b^	6.36 ± 0.4^a^	2.41 ± 0.03^a^	2.46 ± 0.03^a^	0.99 ± 0.03^b^	87 ± 4^b^	27 ± 1^a^ 28 ± 1^a^	7.9 ± 0.3^a^ 5.4 ± 0.4^b^	0.17 ± 0.006^a^ 0.017 ± 0.002^a^	0.13 ± 0.001^a^ 0.016 ± 0.001^b^	0.46 ± 0.001^a^ 0.55 ± 0.01^a^	8.56 ± 0.01^a^ 7.4 ± 0.02^b^
Mix-22	9.0 ± 2^d^	3.04 ± 0.8^b^	1.21 ± 0.01^b^	1.48 ± 0.01^b^	1.64 ± 0.01^a^	91 ± 2^a^	27 ± 1^a^ 28 ± 1^a^	7.9 ± 0.3^a^ 6.7 ± 0.6^a^	0.17 ± 0.006^a^ 0.020 ± 0.004^a^	0.13 ± 0.001^a^ 0.018 ± 0.002^b^	0.46 ± 0.001^a^ 0.55 ± 0.02^a^	8.56 ± 0.01^a^ 7.7 ± 0.01^a^
Mix-28	17 ± 3^b^	6.22 ± 0.3^a^	2.41 ± 0.03^a^	2.46 ± 0.03^a^	0.99 ± 0.03^b^	85 ± 3.5^b^	27 ± 1^a^ 28 ± 1^a^	7.9 ± 0.3^a^ 5.5 ± 0.4^b^	0.17 ± 0.006^a^ 0.018 ± 0.003^a^	0.13 ± 0.001^a^ 0.017 ± 0.001^b^	0.46 ± 0.001^a^ 0.56 ± 0.02^a^	8.56 ± 0.01^a^ 7.3 ± 0.01^b^

^a^ Starch-22, starch carbon source at 22 °C; Starch-28, starch carbon source at 28 °C; Seaweed-22, seaweed carbon source at 22 °C; Seaweed-28; seaweed carbon source at 28 °C; Mix-22, starch–seaweed mix carbon sources at 22 °C; Mix-28, starch–seaweed mix carbon sources at 28 °C. For each water parameter, lowercase letters (a, b, and c) compare values separately. Initial values (top row in each cell) are compared only to other initial values across all treatments. Final values (bottom row) are compared only to other final values. Within each group (initials or finals), values sharing the same letter are not significantly different (*p* < 0.05, Duncan’s test).

### 3.2 BFT molecules profile

**FIGURE 2:**
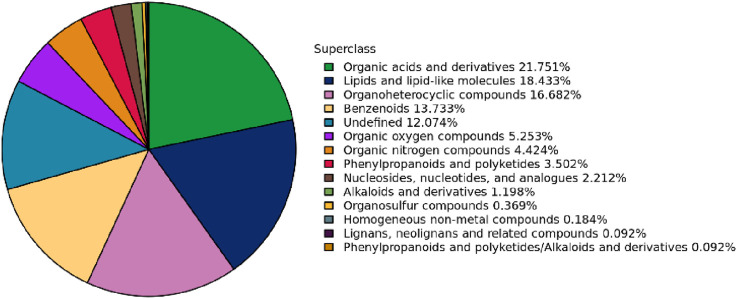
Chemical superclasses from biofloc with *Penaeus vannamei* juveniles after 2 months’ growth with different carbon sources and temperatures.

The most dominant molecules are shown and classified as follows: organic acids, lipids, organoheterocyclic compounds, and benzenoids (Figure 2). Among the treatments, these molecules showed different levels across treatments (Table 3). Seaweed treatment at 22 °C showed the highest level of lipid content, including lauric, hyocholic, and chenodeoxycholic acids; starch-28 showed the highest level of benzenoids, while Mix-28 showed the highest level of organic acids and organoheterocyclic compounds. A total of 7,467 molecules were revealed, and a total of 303 metabolites were identified. Among the metabolites identified, 37 were selected as effective metabolites based on obvious fold-change differences among treatments (Yang et al., 2021), where +3 fold-changes and −4 fold-changes were used the lower limits for selection.

**TABLE 3:** Major chemical superclasses in treatments of biofloc with *Penaeus vannamei* juveniles after 2 months’ growth with different carbon sources and temperatures.

Chemical superclass (%±SE)^a^	T1	T2	T3	T4	T5	T6
Benzenoids	17 ± 1.2	22 ± 1.5	13 ± 1.0	16 ± 1.3	13 ± 1.0	20 ± 1.5
Lipids and lipid-like molecules: (a) lauric acid (b) hyocholic acid (c) chenodeoxycholic acid	25 ± 1.0 92 ± 2.0 22 ± 1.5 22 ± 1.5	15 ± 1.0 1.0 ± 0.2 19 ± 1.3 15 ± 1.2	20 ± 2.0 1.0 ± 0.2 12 ± 1.0 15 ± 1.4	16 ± 2.0 1.0 ± 0.2 9.0 ± 0.8 13 ± 1.1	18 ± 2.0 1.0 ± 0.2 14 ± 1.2 16 ± 1.3	5.5 ± 1.0 4.0 ± 0.5 23 ± 1.5 19 ± 1.5
Organic acids and derivatives	16 ± 1.2	19 ± 1.5	12 ± 1.0	12 ± 1.0	11 ± 0.8	29 ± 2.0
Organoheterocyclic compounds	18 ± 1.2	21 ± 1.6	11 ± 1.0	12 ± 1.0	13 ± 1.0	24 ± 1.5

^a^ Molecule percentage among different treatments. T1 (Starch-22), starch carbon source at 22 °C; T2 (Starch-28), starch carbon source at 28 °C; T3 (Seaweed-22), seaweed carbon source at 22 °C; T4 (Seaweed-28), seaweed carbon source at 28 °C; T5 (Mix-22), starch–seaweed mix carbon sources at 22 °C; and T6 (Mix-28), starch–seaweed mix carbon sources at 28 °C.

**FIGURE 3:**
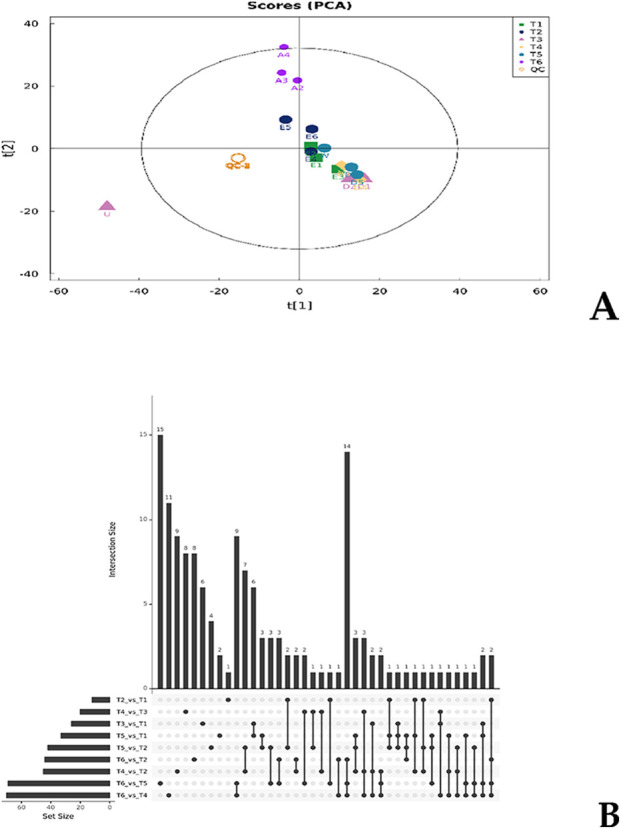
Principal coordinate analysis (PCoA) in biofloc with *Penaeus vannamei* juveniles **(A)** and numbers of shared and unique KO assignments **(B)** after a 2-month growing period with different carbon sources and temperatures based on positive electrospray ionization, n = 3. T1 (Starch-22), starch carbon source at 22 °C; T2 (Starch-28), starch carbon source at 28 °C; T3 (Seaweed-22), seaweed carbon source at 22 °C; T4 (Seaweed-28), seaweed carbon source at 28 °C; T5 (Mix-22), starch–seaweed mix carbon sources at 22 °C; T6 (Mix-28), starch–seaweed mix carbon sources at 28 °C.

Principal coordinate analysis (PCoA) revealed differences between treatments based on distance. Seaweed-22 and Mix-22 showed a short distance; starch at both 22 °C and 28 °C did not show a long distance, while the starch–seaweed mix showed the longer distance at 28 °C compared to 22 °C (Figure 3). The intersection between treatments was wide: Mix-28 showed the highest molecule level (L-carnitine, LCA) compared to Mix-22 (450 fold-changes), Seaweed-28 (250 fold-changes), and Starch-28 (approximately 20 fold-changes). Seaweed treatments increased the telmisartan level compared to starch treatments by three fold-changes at both temperatures. Mix-28 increased mesaconic acid by 12 fold-changes compared to Mix-22, while Mix-22 increased isobutyric acid about six fold-changes compared to Starch-22 and Starch-28 (Figure 3).

#### 3.2.1 Bacterial activity activators

Biofloc molecules exhibited variation among treatments, including carbon sources, nitrogen sources, electron acceptors, stress mediators, and bacterial metabolites (Table 4). The profile of different molecules directly influenced bacterial communities by affecting the availability of carbon and nitrogen nutrients. In contrast, antioxidants and metabolic byproducts exerted indirect effects on these communities. This is because antioxidants positively impact metabolic systems, while metabolic byproducts can have either positive or negative effects on different bacterial metabolic processes. Notably, some molecules have been recognized for their effects on bacterial communities, while others have no clear function in the bacterial metabolic system in the scientific literature.

**TABLE 4:** Bacterial activity activator molecule profiles in a 2-month growing period of *Penaeus vannamei* biofloc system with different carbon sources and temperatures.

Treatment comparison	Molecule name	Fold-changes^a^ (log_2_)	Biological effect	Detected bacteria associated with bioactive molecules (literature-based)	Reference
Seaweed-22 vs. Starch-22	Didodecyl 3,3′-thiodipropionate oxide	12.0	Antioxidant	General effect	Beckmann et al. (2021), Weber et al. (2006)
Seaweed-28 vs. Starch-28		12.0			
Seaweed-22 vs. Starch-22	16-Oxocafestol	11.0	—	—	—
Seaweed-28 vs. Starch-28		11.0			
Seaweed-28 vs. Starch-28	Telmisartan	3.00	—	Firmicutes/Bacteroidota Blautina Allobaculum Parasutterella	Beckmann et al. (2021)
Seaweed-28 vs. Starch-28	Isethionate	−6.0	Byproduct of taurine oxidation by bacteria	*Ruegeria pomeroyi*	Burrichter et al. (2021), Kertesz (2000), Weinitschke et al. (2010)
Seaweed-28 vs. Seaweed-22	Quinate	−6.0	Carbon source	*Corynebacterium glutamicum*	Teramoto et al. (2009)
Mix-22 vs. Starch-22	Isobutyric acid	6.00	Bacterial by-product	*Vibrio* Cyanobacteria	Kumar et al. (2013), Tokuda et al. (1982), Zhang et al. (2011)
Mix-22 vs. Starch-28	Vitamin K1	3.50	Bacterial by-product, Cardinal redox cofactor	Cyanobacteria	Klughammer and Pace (1997), Oostende et al. (2011)
Mix-22 vs. Starch-28	Picric acid	−4.5	Substrate for bacteria	*Pseudomonas aeruginosa* *Rhodococcus erythropolis* *Nocardioides simplex*	Rajan et al. (1996)
Mix-22 vs. Starch-28	3-Methyloxyindole	−4.0	Bacterial by-product	General effect	Gray and Squires (2013), Li et al. (2021)
Mix-22 vs. Starch-28	Melibiose	−9.0	Carbon source, disaccharide	General effect	Guan and Hariharan (2021), Katsube et al. (2021)
Mix-28 vs. Starch-28	L-carnitine	30.0	Carbon source, nitrogen source, electron acceptor, compatible solute	*Pseudomonas aeruginosa* *P. putida* *P. syringae* *Tetragenococcus halophilus* *Lactobacillus plantarum* *Bacillus subtilis* *Listeria* (pathogenic bacteria) *Xanthomonas translucens* *Burkholderia cepacia* *Rhizobium* sp. *Agrobacterium* sp. *Enterobacterium* sp. *Sinorhizobium meliloti* *Serratia marcescens* *Actinobacter calcoaceticus* *Actinobacter baumannii* Beta proteobacteria (Achromo bacteria) Firmicutes (Sporosarcina) *Klebsiella pneumoniae* *Escherichia coli* *Citrobacter* Providencia *Shigella* *Salmonella typhimurium* *Proteus* spp.	Ghonimy et al. (2018), Meadows and Wargo (2015)
Mix-28 vs. Mix-22		450			
Mix-28 vs. Mix-22		250			
Starch-28 vs. Starch-22	Prosulfocarb	17.0	—	*Pseudomonas* Consortium	Gennari et al. (2002)
Mix-28 vs. Starch-28	1,4-D-xylobiose	51.0	Complex sugar	Bifidobacteria *Lactobacillus*	Zhang (2014)
Mix-28 vs. Mix-22		32.0			

^a^ Fold-change calculated using the formula log_2_ (X/Y). X and Y refer to different treatments: Starch-22, starch carbon source at 22 °C; Starch-28, starch carbon source at 28 °C, Seaweed-22, seaweed carbon source at 22 °C; Seaweed-28, seaweed carbon source at 28 °C; Mix-22, starch–seaweed mix carbon sources at 22 °C; Mix-28, starch–seaweed mix carbon sources at 28 °C. Log_2_ fold change values were calculated using the mean metabolite abundance for each group as log_2_ (mean X/mean Y).

#### 3.2.2 Bacterial activity inhibitors

Biofloc molecules showed differences between treatments regarding bacterial physiology. These molecules included antibiotic metabolites, antibacterial agents, and antibacterial lipopolysaccharide (LPS) (Table 5). Antibiotic molecules may originate from feed, water sources, or bacteria, whereas antibacterial agents can be derived from herbal feed additives commonly used in China’s aquaculture industry. Additionally, the fiber content in feed residue can serve as a source of complex sugars, which may negatively impact the growth of certain bacteria. Other molecules, on the other hand, may have a retardant effect on bacterial growth.

**TABLE 5:** Bacterial activity inhibitor molecule profiles in a 2-month growing period of *Penaeus vannamei* biofloc system with different carbon sources and temperatures.

Treatment comparison	Molecule name	Fold-changes^a^ (log_2_)	Biological effect	Detected bacteria associated with bioactive molecules (literature-based)	Reference
Starch-28 vs. Starch-22	Desethylene ciprofloxacin	−11	Byproduct of antibiotic action	—	Zotou and Miltiadou (2002)
Seaweed-22 vs. Starch-22		−35			
Mix-22 vs. Starch-22		−17			
Seaweed-28 vs. Starch-28		−35			
Starch-28 vs. Starch-22	Probucol	−10	Affects bacterial LPS	Gram-negative bacteria (pathogenic bacteria)	Zucoloto et al. (2017)
Seaweed-28 vs. Starch-28		−6.0			
Starch-28 vs. Starch-22	Prosulfocarb	17.0	—	—	Gennari et al. (2002)
Seaweed-28 vs. Seaweed-22	Tebuconazole	11.0	—	Nitrifying bacteria Denitrifying bacteria Nitrogen fixation bacteria *Anguillospora longissima* Pestalotiopsis Cyanobacteria	Cycoń et al. (2006), Dimitrov et al. (2014); Ortiz-Cañavate et al. (2019)
Mix-22 vs. Starch-22	Fosetyl	−32	Antibacterial agent	*Xylella fastidiosa*	Baldassarre et al. (2020)
Mix-28 vs. Mix-22		65.0			
Mix-22 vs. Starch-22	4-Piperidinecarboxamide	−5.0	Antibacterial agent	*Candida tropicalis* *C. albicans* *Staphylococcus epidermidis*	Tuyun et al. (2019)
Mix-22 vs. Starch-28	Sulfamethazine	−28	Antibiotic	*Paenarthrobacter ureafaciens*	Ou et al. (2015), Yu et al. (2020)
Mix-22 vs. Starch-28	Rosmarinate	−14	Antibacterial agent	*Staphylococcus carnosus* *E. coli*	Suriyarak et al. (2013)
Mix-22 vs. Starch-28	Cyclovirobuxine d	6.00	Antibacterial agent	General effect	Capelli et al. (2021), Yan et al. (2011)
Mix-28 vs. Starch-28	Irisflorentin	54.0	Anti-lipopolysaccharide (Anti-LPS)	General effect	Gao et al. (2014)
Mix-28 vs. Starch-28	Galactinol	59.0	Antibacterial agent	Rhizobacterium *Pseudomonas chlororaphis* E. corotovora	Cho et al. (2010)
Mix-28 vs. Mix-22		22.0			
Mix-28 vs. Starch-28	1,4-D-xylobiose	51.0	Antibacterial agent, complex sugar	General effect	Zhang (2014)
Mix-28 vs. Mix-22		32.0			
Mix-28 vs. Mix-22	Propranolol	250	Decreases the over bacterial growth	*Streptococcus mutans* (pathogenic)	Pérez-Paramo et al. (2000)

^a^ Fold-change calculated using formula log_2_ (X/Y). X and Y refer to different treatments: Starch-22, starch carbon source at 22 °C; Starch-28, starch carbon source at 28 °C; Seaweed-22, seaweed carbon source at 22 °C; Seaweed-28; seaweed carbon source at 28 °C; Mix-22, starch–seaweed mix carbon sources at 22 °C; Mix-28, starch–seaweed mix carbon sources at 28 °C. Log_2_ fold change values were calculated using the mean metabolite abundance for each group as log_2_ (mean X/mean Y).

#### 3.2.3 Xenobiotics

The profile of biofloc molecules showed different levels of xenobiotics, including anti-algae, pesticides, anti-fungal agents, drugs, and hormone molecules (Table 6). Anti-algal, pesticidal, and human metabolic molecules are primarily derived from water sources. Essential oils and medical drugs are largely broken down by BFT bacteria, resulting in the formation of various molecules. There is a complex interplay between the sources of these molecules and the bacterial community within the BFT system.

**TABLE 6:** Xenobiotic profiles in a 2-month growing period of *Penaeus vannamei* juveniles in the biofloc system with different carbohydrate sources and temperatures.

Treatment comparison	Molecule name	Fold-changes^a^ (log_2_)	Biological effect	Detected bacteria associated with bioactive molecules (literature-based)	Reference
Starch-28 vs. Starch-22	2-Ethylhexyl diphenyl phosphate	−85	—	—	—
Starch-28 vs. Starch-22	Mandipropamid	21.0	Anti-algal	Decrease Cyanobacteria	Blum et al. (2010)
Seaweed-22 vs. Starch-22	Imazethapyr	37.0	Pesticide	Decrease Rhizobium	Singh and Singh (2020)
Seaweed-28 vs. Starch-28		37.0			
Seaweed-28 vs. Seaweed-22	2-Methoxy-5-nitrophenol	3.50	Anti-fungal, anti-bacterial, eugenol derivative	*Klebsiella pneumoniae*	Prichard (1973), Wang et al. (2019)
Mix-22 vs. Starch-22	16-alpha-hydroxyestrone	14.0	—	—	—
Mix-22 vs. Starch-28	Cytidine	−4.0	Bacterial substrate	*E. coli* *Bacillus subtilis*	Navaratnam and Sarwar (2006)
Mix-28 vs. Starch-28	5-Methylisoxazol-3-amine	−22	By-product of drug oxidation by bacteria	​	Monier et al. (2018)
Mix-28 vs. Starch-28	1-heptanamine	37.0	—	—	—
Mix-28 vs. Mix-22	Tetradecylphosphonate	14.0	—	—	Mohammadi Ziarani et al. (2019)
Mix-28 vs. Seaweed-28	Prostaglandin F_2β_	31.0	Human metabolite	—	—
Mix-28 vs. Seaweed-28	Cholesteryl sulfate	−32	Anti-fungal	—	—
Mix-28 vs. Seaweed-28	Pentadecanoic sulfate	−21	Bacterial substrate	—	Callaghan et al. (2006)
Mix-28 vs. Mix-22	Mesaconic acid	12.0	Bacterial substrate	*Pseudomonas*	Brightman and Martin (1961)

^a^ Fold-change was calculated using the formula of log_2_ (X/Y). X and Y refer to different treatments; Starch-22, starch carbon source at 22 °C; Starch-28, starch carbon source at 28 °C; Seaweed-22, seaweed carbon source at 22 °C; Seaweed-28, seaweed carbon source at 28 °C; Mix-22, starch–seaweed mix carbon sources at 22 °C; and Mix-28, starch–seaweed mix carbon sources at 28 °C. Log_2_ fold change values were calculated using the mean metabolite abundance for each group as log_2_ (mean X/mean Y).

#### 3.2.4 Secondary metabolites

Other identified metabolites were related to bacteria–bacteria interaction, bacterial secondary metabolites, and carbon source phenolic metabolites (Table 7). The growth of heterotrophic bacteria was identified through the presence of a common metabolite, which serves as a chemical indicator characterizing the microbiota in the BFT system. LCA, a distinct indicator primarily derived from animal-based proteins, is partially consumed by intestinal bacteria under certain physiological conditions, while the remaining portion is excreted into the BFT environment. This makes LCA a potential chemical indicator of bacterial activity and physiological status in the intestinal environment. Additionally, phenolic compounds are known for their antimicrobial effects against pathogenic bacteria, with different phenols exhibiting varying effects at different concentrations. These compounds could also serve as indicators of the physiological status of the bacterial community.

**TABLE 7:** Specific bioactive molecule profiles in a 2-month growing period with *Penaeus vannamei* juveniles in the biofloc system with different carbohydrate sources and temperatures.

Classification	Metabolite name	Treatments comparisons (fold-changes)^a^			
		Seaweed-22 vs. Starch-22	Mix-22 vs. Starch-22	Seaweed-22 vs. Starch-28	Mix-28 vs. Starch-28
Heterotrophic by-product	2-(2′,3′,4′-Trihydroxybutyl)quinoxaline	6.70	7.90	2.400	2.500
L-carnitine metabolite	Trimethylamine	0.20	0.57	0.300	0.560
	Pyruvate	0.30	0.17	0.028	0.034
Phenolic compound	Benzenoids	0.55	0.80	0.690	4.200
	2-Aminophenol	1.48	0.98	0.980	0.790
	2-Propylphenol	0.26	0.75	0.640	2.470
	Cinnamylphenol	0.88	2.27	0.260	0.520
	Methoxyphenol	0.31	0.31	0.620	2.370
	1-Hydroxy-2-unsubstituted benzenoids	0.44	0.48	0.420	0.770
	Anisoles	4.80	0.64	0.005	0.006
	Bisphenol	0.61	0.81	0.720	0.330

^a^ Starch-22, starch carbon source at 22 °C; Starch-28, starch carbon source at 28 °C; Seaweed-22, seaweed carbon source at 22 °C; Seaweed-28, seaweed carbon source at 28 °C; Mix-22, starch–seaweed mix carbon sources at 22 °C; Mix-28, starch–seaweed mix carbon sources at 28 °C. Log_2_ fold change values were calculated using the mean metabolite abundance for each group as log_2_ (mean X/mean Y).

### 3.3 Microbiota composition and bacterial diversity in shrimp intestines

**FIGURE 4:**
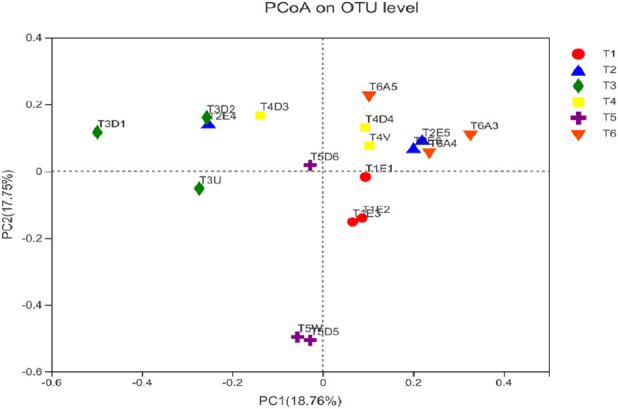
Principal coordinate analysis (PCoA) from intestinal tract of shrimp (*Penaeus vannamei*) juveniles after a 2-month growing period in BFT with different carbon sources and temperatures. T1 (Starch-22), starch carbon source at 22 °C; T2 (Starch-28), starch carbon source at 28 °C; T3 (Seaweed-22), seaweed carbon source at 22 °C; T4 (Seaweed-22), seaweed carbon source at 28 °C; T5 (Mix-22), starch–seaweed mix carbon sources at 22 °C; T6 (Mix-28), starch–seaweed mix carbon sources at 28 °C.

All 28 °C treatments showed shorter distances among each other than the 22 °C treatments, while 22 °C treatments showed greater distances between each other (Figure 4). The cumulative variance of 36.51% explained by PC1 and PC2 is acceptable for exploratory analysis in microbiome studies, where the minimal acceptable cumulative variance is explained by PC1 and PC2 for microbiome studies larger than 30%. The key finding is that the PCA plot shows a clear separation of samples based on the experimental treatments (carbon source and temperature). This indicates that the primary treatment-driven biological variation in the system was successfully captured by these components.

#### 3.3.1 Bacterial community indices

Phylotype richness was estimated according to the bias-corrected Chao1 estimator calculation. Chao1 scores ranged 489–599 for 22 °C treatments and 552–688 for 28 °C treatments (Table 8). Interestingly, Seaweed-22 and Starch-28 showed a higher phylotype richness than the other 22 °C and 28 °C treatments, respectively (Table 9). The bacterial α-diversity (Shannon index) was higher in Starch-28 than in other 28 °C treatments, whereas 22 °C treatments did not show a significant difference (Table 9). The Simpson index showed the highest value for Seaweed-28 compared to other 28 °C treatments, whereas no significant differences (*p* > 0.05) between 22 °C treatments were clarified (Table 9).

**TABLE 8:** Bacterial diversity in intestine of *Penaeus vannamei* juveniles after a 2-month growing period in biofloc system with different carbon sources and temperatures.

Treatment^a^	Chao1 index (±SE)	Shannon index (±SE)	Simpson index (±SE)
Starch-22	553 ± 20^b^	4.0 ± 0.2^a^	0.03 ± 0.01^c^
Starch-28	688 ± 30^a^	4.2 ± 0.2^a^	0.04 ± 0.01^c^
Seaweed-22	599 ± 9^a^	4.0 ± 0.2^a^	0.03 ± 0.01^c^
Seaweed-28	55,219 ± ^c^	3.1 ± 0.2^c^	0.1 ± 0.01^a^
Mix-22	489 ± 12^c^	3.9 ± 0.2^ab^	0.04 ± 0.01^c^
Mix-28	576 ± 26^b^	3.6 ± 0.2^b^	0.09 ± 0.02^b^

Superscript letters indicate significant differences among treatment within each column (*p* ≤ 0.05); the statistical analysis was performed independently for each temperature group.

^a^ Starch-22, starch carbon source at 22 °C; Starch-28, starch carbon source at 28 °C; Seaweed-22, seaweed carbon source at 22 °C; Seaweed-28, seaweed carbon source at 28 °C; Mix-22, starch–seaweed mix carbon sources at 22 °C; Mix-28, starch–seaweed mix carbon sources at 28 °C.

**TABLE 9:** Relative abundance of bacterial phyla identified in shrimp (*Penaeus vannamei)* intestine reared in biofloc technology (BFT) with different carbon sources.

Phylum	Treatment^a^					
	Starch-22 (±SE)	Starch-28 (±SE)	Seaweed-22 (±SE)	Seaweed-28 (±SE)	Mix-22 (±SE)	Mix-28 (±SE)
Proteobacteria	0.422 ± 0.035^b^	0.677 ± 0.040^a^	0.551 ± 0.038^ab^	0.381 ± 0.032^c^	0.466 ± 0.036^b^	0.719 ± 0.042^a^
Bacteroidota	0.229 ± 0.025^a^	0.063 ± 0.010^c^	0.162 ± 0.02^b^	0.295 ± 0.028^a^	0.103 ± 0.015^bc^	0.111 ± 0.016^bc^
Actinobacteriota	0.230 ± 0.025^a^	0.123 ± 0.018^b^	0.197 ± 0.022^a^	0.061 ± 0.012^c^	0.206 ± 0.023^a^	0.085 ± 0.014^bc^
Firmicutes	0.037 ± 0.008^a^	0.019 ± 0.006^ab^	0.024 ± 0.007^ab^	0.209 ± 0.023^c^	0.034 ± 0.023^a^	0.027 ± 0.007^ab^
Verrucomicrobiota	0.045 ± 0.009^a^	0.03 ± 0.007^a^	0.017 ± 0.006^ab^	0.001 ± 0.001^b^	0.079 ± 0.014^c^	0.002 ± 0.001^b^
Chloroflexi	0.015 ± 0.005^a^	0.042 ± 0.009^a^	0.032 ± 0.008^ab^	0.033 ± 0.008^ab^	0.086 ± 0.014^c^	0.034 ± 0.008^ab^
Patescibacteria	0.008 ± 0.003^b^	0.017 ± 0.005^a^	0.007 ± 0.003^ab^	0.006 ± 0.003^ab^	0.002 ± 0.001^b^	0.006 ± 0.003^ab^
Dependentiae	0.003 ± 0.001^b^	0.006 ± 0.002^b^	0.001 ± 0.000^b^	0.004 ± 0.002^b^	0.011 ± 0.004^b^	0.001 ± 0.001^b^
Firmicutes/Bacteroidota	0.160 ± 0.025^c^	0.300 ± 0.035^b^	0.140 ± 0.022^c^	0.700 ± 0.45^a^	0.330 ± 0.038^b^	0.240 ± 0.030^bc^

^a^ Starch-22, starch carbon source at 22 °C; Starch-28, starch carbon source at 28 °C; Seaweed-22, seaweed carbon source at 22 °C; Seaweed-28, seaweed carbon source at 28 °C; Mix-22, starch–seaweed mix carbon sources at 22 °C; Mix-28, starch–seaweed mix carbon sources at 28 °C.

#### 3.3.2 Microbiota composition

**FIGURE 5:**
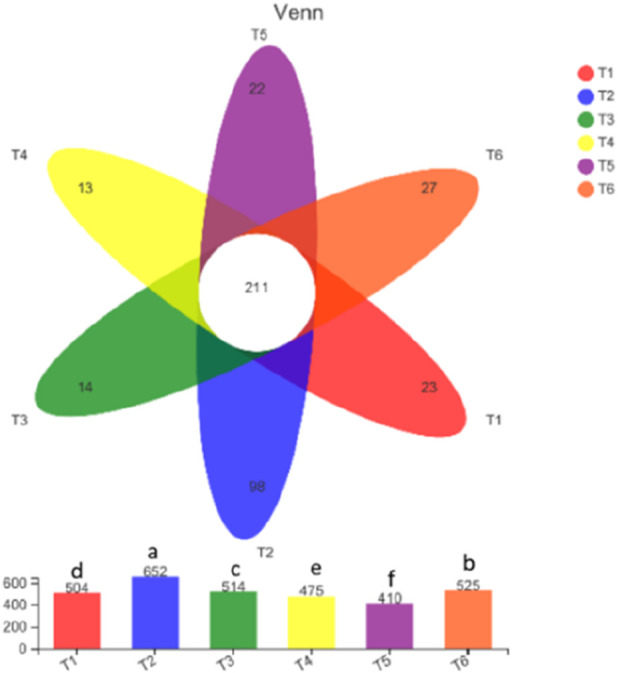
Venn diagram showing the shared OTUs of intestine bacteria of shrimp (*Penaeus vannamei*) juveniles after a 2 months’ growth in BFT with different carbon sources and temperatures. T1 (Starch-22), starch carbon source at 22 °C; T2 (Starch-28), starch carbon source at 28 °C; T3 (Seaweed-22), seaweed carbon source at 22 °C; T4 (Seaweed-28), seaweed carbon source at 28 °C; T5 (Mix-22), starch–seaweed mix carbon sources at 22 °C; T6 (Mix-28), starch–seaweed mix carbon sources at 28 °C.

In this study, the starch, seaweed, and their mix were applied to the biofloc system, which showed an effect on the microbiota composition and *vibrio* abundance in culture water. Venn diagram analyses showed significant differences in the frequency distribution of bacterial operational taxonomic units (OTUs) based on the effect of carbon sources at 28 °C temperature, whereas the Starch-22 or Mix-22 showed a higher significant difference than Seaweed-22 (Figure 5).

**FIGURE 6:**
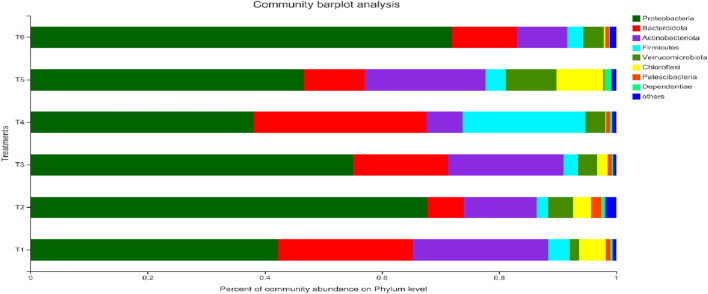
Relative abundance of bacterial phyla determined in shrimp (*Penaeus vannamei*) intestine after 2 months’ growth in biofloc with different carbon sources and temperatures. T1 (Starch-22), represent the starch carbon source treatment at 22 °C; T2 (Starch-28), represents the starch carbon source treatment at 28 °C; T3 (Seaweed-22), represents the seaweed carbon source treatment at 22 °C; T4 (Seaweed-28), represents the seaweed carbon source treatment at 28 °C; T5 (Mix-22), represents the starch–seaweed mix carbon sources treatment at 22 °C; T6 (Mix-28), represents the starch–seaweed mix carbon sources treatment at 28 °C.

At the phylum level, the 22 °C treatments showed the highest abundance of Bacteroidota in Starch-22, Verrucomicrobiota, Chloroflexi, and Dependentiae in Mix-22, and Actinobacteriota abundance was similar in all 22 °C treatments (Figure 6; Table 9). Bacteroidota/Firmicutes ratio was highest in Mix-22 and Seaweed-28 compared to the other 22 °C and 28 °C treatments, respectively (Table 9).

**FIGURE 7:**
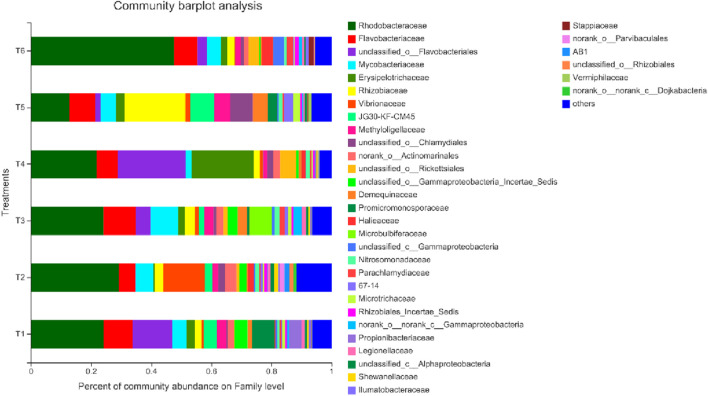
Relative abundance of bacterial family determined in shrimp (*Penaeus vannamei)* intestine after 2 months’ growth in biofloc with different carbon sources and temperatures. T1 (Starch-22), starch carbon source at 22 °C; T2 (Starch-28), starch carbon source at 28 °C; T3 (Seaweed-22), seaweed carbon source at 22 °C; T4 (Seaweed-28), seaweed carbon source at 28 °C; T5 (Mix-22), starch–seaweed mix carbon sources at 22 °C; T6 (Mix-28), starch–seaweed mix carbon sources at 28 °C.

**FIGURE 8:**
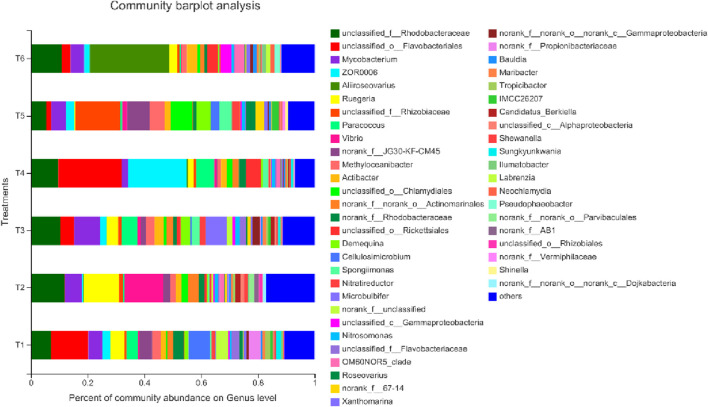
Relative abundance of bacterial genera determined in shrimp (*Penaeus vannamei)* intestine after 2 months’ growth in biofloc with different carbon sources and temperatures.

At family and genus levels, *Vibrio* showed increased abundances in Mix-22 and Starch-28 compared to the other 22 °C and 28 °C treatments, respectively, and decreased abundances in Starch-22 and Mix-28 compared to the other 22 °C and 28 °C treatments, respectively (Figures 7, 8; Tables 10, 11). *Rhizobium* showed the highest abundance at Starch-28 (family and genus) and Mix-28 (family) compared to the other 28 °C treatments (Figures 7, 8; Tables 10 and 11). *Xanthomarina* showed the highest abundance at Starch-22 and Mix-28 at genus level compared to the other 22 °C and 28 °C treatments, respectively (Figure 8; Table 11).

**TABLE 10:** Relative abundance of bacterial families identified in shrimp (*Penaeus vannamei*) intestine reared in biofloc technology (BFT) with different carbon sources.

Family	Treatment^a^					
	Starch-22 (±SE)	Starch-28 (±SE)	Seaweed-22 (±SE)	Seaweed-28 (±SE)	Mix-22 (±SE)	Mix-28 (±SE)
Rhizobiaceae	0.022 ± 0.007^b^	0.028 ± 0.008^b^	0.034 ± 0.010^b^	0.019 ± 0.006^b^	0.201 ± 0.060^a^	0.019 ± 0.006^b^
Rhizobiales	0.006 ± 0.002^ab^	0.009 ± 0.003^a^	0.007 ± 0.002^ab^	0.005 ± 0.002^b^	0.007 ± 0.002^ab^	0.014 ± 0.004^a^
Vibrionaceae	0.005 ± 0.002^c^	0.137 ± 0.041^a^	0.034 ± 0.010^b^	0.009 ± 0.003^c^	0.017 ± 0.005^bc^	0.002 ± 0.001^c^
Gammaproteobacteria *2*	0.055 ± 0.017^a^	0.013 ± 0.004^bc^	0.069 ± 0.021^a^	0.010 ± 0.003^c^	0.008 ± 0.002^c^	0.043 ± 0.013^ab^

^a^ Starch-22, starch carbon source at 22 °C; Starch-28, starch carbon source at 28 °C; Seaweed-22, seaweed carbon source at 22 °C; Seaweed-28, seaweed carbon source at 28 °C; Mix-22, starch–seaweed mix carbon sources at 22 °C; Mix-28, starch–seaweed mix carbon sources at 28 °C.

**TABLE 11:** Relative abundance of bacterial genera identified in shrimp (*Penaeus vannamei)* intestine reared in biofloc technology (BFT) with different carbon sources.

Genus	Treatment^a^					
	Starch-22 (±SE)	Starch-28 (±SE)	Seaweed-22 (±SE)	Seaweed-28 (±SE)	Mix-22 (±SE)	Mix-28 (±SE)
*Rhizobiales*	0.000 ± 0.000^b^	0.012 ± 0.006^a^	0.000 ± 0.000^b^	0.000 ± 0.000^b^	0.000 ± 0.000^b^	0.000 ± 0.000^b^
*Vibrio*	0.005 ± 0.002^c^	0.134 ± 0.006^a^	0.012 ± 0.003^b^	0.009 ± 0.002^bc^	0.017 ± 0.004^b^	0.001 ± 0.000^c^
*Gammaproteobacteria*	0.012 ± 0.005^b^	0.005 ± 0.002^c^	0.036 ± 0.008^a^	0.000 ± 0.000^d^	0.008 ± 0.002^c^	0.047 ± 0.010^a^
Rhodobacteraceae *2*	0.037 ± 0.008^a^	0.020 ± 0.004^ab^	0.012 ± 0.003^b^	0.023 ± 0.005^ab^	0.014 ± 0.003^b^	0.011 ± 0.002^b^
*Pseudophaeobacter*	0.000 ± 0.000^a^	0.000 ± 0.000^a^	0.000 ± 0.000^a^	0.000 ± 0.000^a^	0.000 ± 0.000^a^	0.000 ± 0.000^a^
*Xanthomarina* (*Pseudomonas*)	0.011 ± 0.002^ab^	0.003 ± 0.001^c^	0.005 ± 0.001^bc^	0.005 ± 0.001^bc^	0.012 ± 0.003^ab^	0.013 ± 0.003^a^

^a^ Starch-22, starch carbon source at 22 °C; Starch-28, starch carbon source at 28 °C; Seaweed-22, seaweed carbon source at 22 °C; Seaweed-28, seaweed carbon source at 28 °C; Mix-22, starch–seaweed mix carbon sources at 22 °C; Mix-28, starch–seaweed mix carbon sources at 28 °C.

**FIGURE 9:**
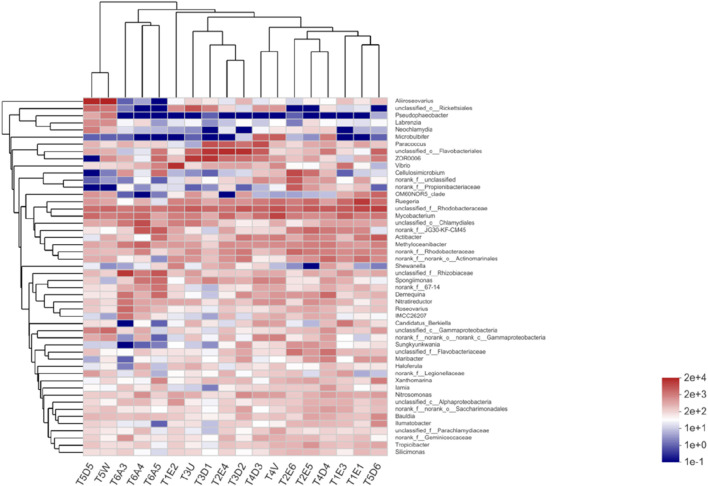
Heatmap of microbial community composition at the genus level. The heatmap uses a color gradient (blue to red) to represent low to high abundance, with the scale ranging from 1 × 10^−1^ to 2 × 10^4^ (as indicated by the color bar). Rows correspond to individual genera, while columns represent different samples (labeled T5D5, T5W, T6A3, *etc.*). T, treatment and its replicates; T1 (Starch-22), starch carbon source at 22 °C; T2 (Starch-28), starch carbon source at 28 °C; T3 (Seaweed-22), seaweed carbon source at 22 °C; T4 (Seaweed-28), seaweed carbon source at 28 °C; T5 (Mix-22), starch–seaweed mix carbon sources at 22 °C; T6 (Mix-28), starch–seaweed mix carbon sources at 28 °C.

**FIGURE 10:**
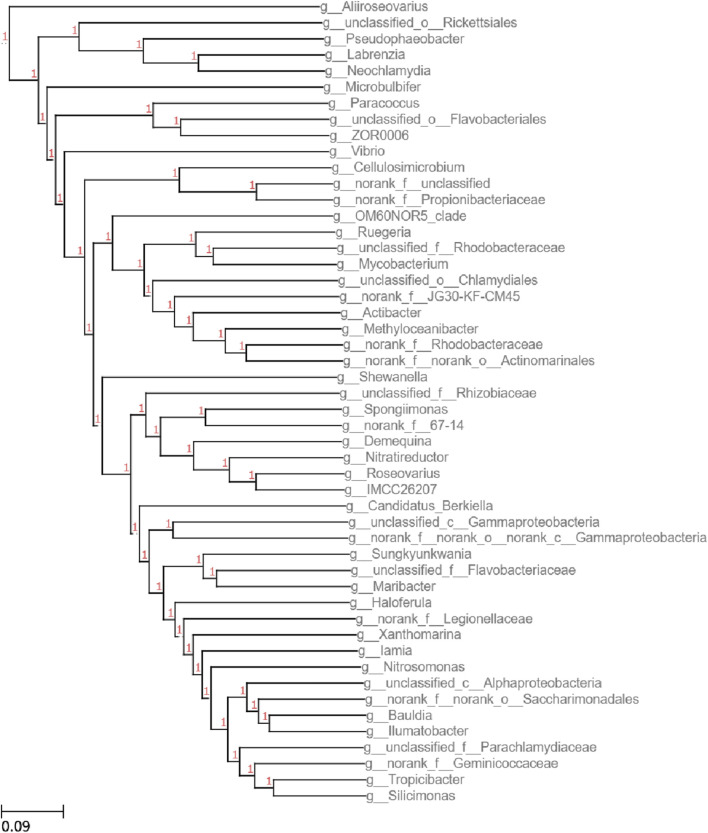
Phylogenetic tree of identified bacterial species, illustrating the evolutionary relationships among various bacterial genera identified in this study. Nodes are labeled with bootstrap values (all shown as “1”), indicating strong support for the branching patterns. The scale bar at bottom left represents a genetic distance of 0.09, providing a reference for the evolutionary divergence between species.

Hierarchical clustering of both rows (genera) and columns (samples) reveals patterns of similarity in community composition. Notably, certain genera (e.g., *Aliiroseovarius* and *Pseudophaeobacter*) exhibit high abundance in specific sample clusters, while others (e.g., unclassified taxa) show more variable or widespread distribution. This visualization highlights the compositional differences and similarities among the microbial communities analyzed, providing insight into the structuring of bacterial populations across samples (Figure 9). The phylogenetic tree helps visualize the phylogenetic clustering and diversity of the bacterial community analyzed. Species such as *g__Aliiroseovarius*, *g__Pseudophaeobacter*, *and g__Silicimonas* are included, with some taxa marked as “unclassified” or “norank,” reflecting incomplete taxonomic annotation (Figure 10).

### 3.4 Functional pathway prediction from the biofloc water metabolome

**FIGURE 11:**
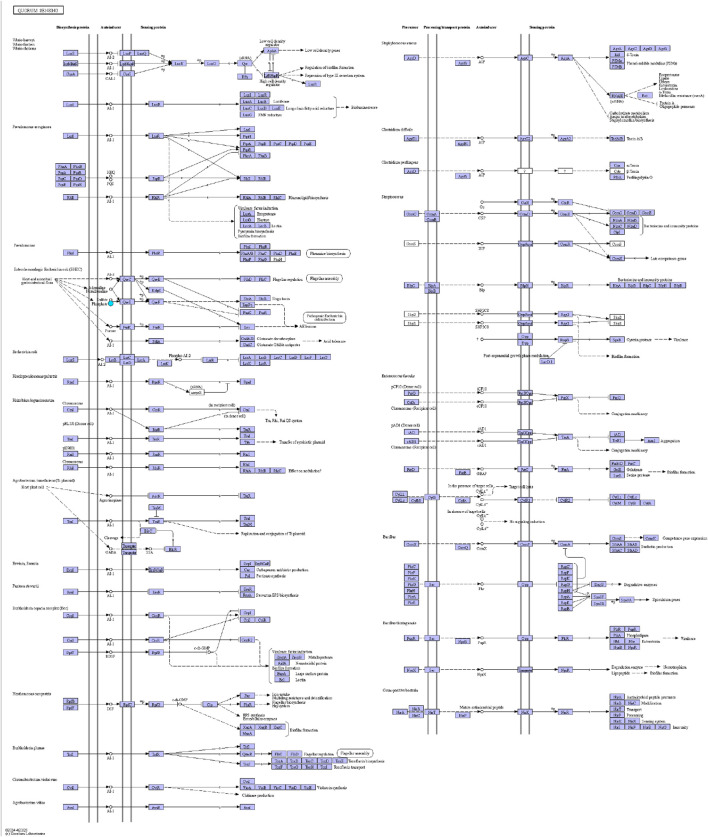
Impact of carbon source and temperature on microbial sensory and regulatory pathways. Comparing T4 versus T2. T2 (Starch-28), starch carbon source at a temperature of 28oC; T4 (Seaweed-28), seaweed carbon source at a temperature of 28°C.

*Pathogenic inhibition*. Functional prediction revealed that the microbial community in the T4 (Seaweed-28 °C) treatment was significantly enriched for pathways related to bacterial communication and environmental sensing compared to other treatments. Specifically, quorum sensing pathways were upregulated *versus* T2 (Starch-28), indicating an enhanced potential to interfere with pathogenic virulence regulation. Concurrently, two-component system pathways were enriched in both T4 and T3 (Seaweed-22) *versus* T1 (Starch-22), suggesting that the seaweed carbon source reprograms microbial sensory networks for better environmental adaptation and pathogen resilience (Figure 11).

**FIGURE 12:**
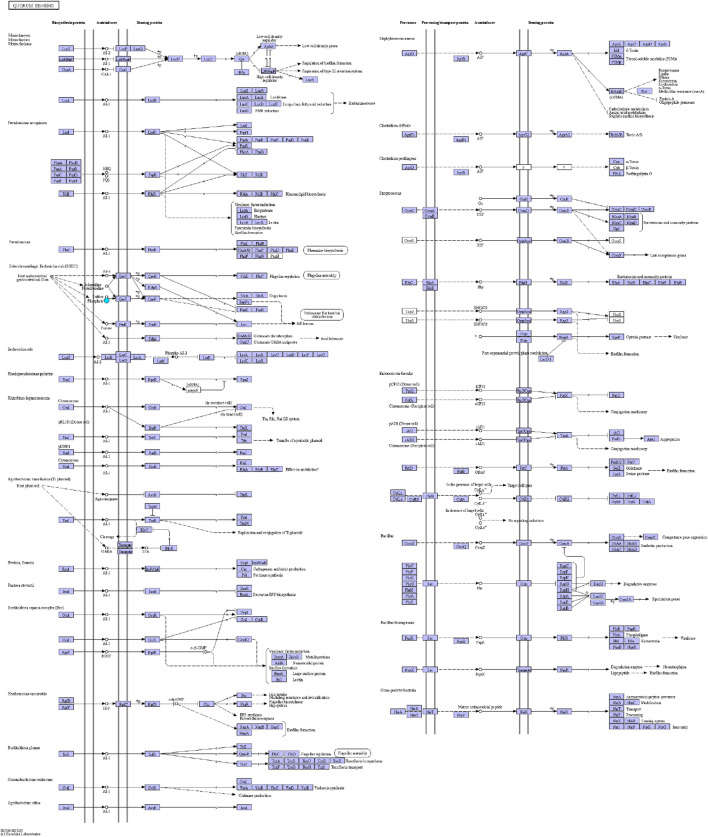
Impact of Carbon Source and Temperature on butanoate metabolism. Comparing T6 versus T5. T5 (Mix-22), starch-seaweed mix carbon sources at a temperature of 22oC; and T6 (Mix-28), starch-seaweed mix carbon sources at a temperature of 28°C.

*Nutrient Metabolism*. The analysis highlighted distinct metabolic profiles driven by carbon source and temperature. The T4 treatment showed enriched pathways for nitrogen and carbon metabolism compared to T2, supporting efficient nutrient cycling. Furthermore, oxidative phosphorylation was enhanced in seaweed-based treatments (T4 vs. T2; T3 vs. T1), indicating higher energy production. Notably, the optimal Mix-28 (T6) treatment exhibited a significant upregulation in butanoate metabolism *versus* Mix-22 (T5), pointing to an increased potential for beneficial short-chain fatty acid production that supports host gut health (Figure 12).

**FIGURE 13:**
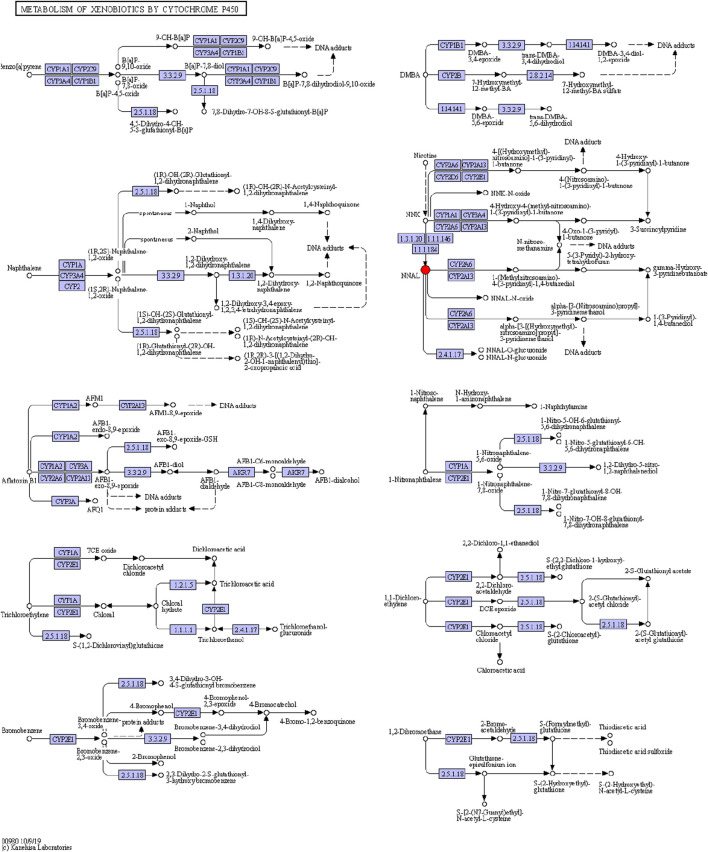
Impact of carbon source and temperature on xenobiotic biodegradation pathways. Comparing T4 versus T3. T3 (Seaweed-22), seaweed carbon source at a temperature of 22oC; T4 (Seaweed-28), seaweed carbon source at a temperature of 28oC.

*Xenobiotic Metabolism*. The microbial community in the T4 treatment also demonstrated a heightened capacity for processing foreign compounds. Pathways for xenobiotic biodegradation were enriched *versus* both T2 and T3, and drug metabolism pathways were specifically upregulated *versus* T3. This suggests that the combined conditions of seaweed carbon source and 28 °C temperature select for a microbiome with a robust detoxification potential, likely contributing to system stability by degrading complex organic pollutants (Figure 13).

## 4 Discussion

### 4.1 Biofloc volume, growth performance, survival rate, and water chemical parameters

The chemical composition of BFT was influenced by water temperature and carbon source, which, in turn, impacted the microbial composition of shrimp intestine through digested bioflocs. Treatments involving starch-22 and mix-28 demonstrated lower *Vibrio* abundance and higher levels of commensal bacteria in shrimp intestine than other treatments under varying temperature conditions. This effect may be associated with the increased levels of telmisartan, a compound known to enhance commensal bacterial populations (Beckmann et al., 2021) and bile acids (pathogenic bacterial control) (Cahill, 1983; Glatman-Freedman et al., 2000; Kocsár et al., 1969; Van Bossuyt et al., 1988); it is indicated by the elevated levels of LCA (an indicator for less stressed bacterial community) and mesaconic acid (byproduct of astaxanthin producing bacteria) (Brightman and Martin, 1961; Ghonimy et al., 2018; Meadows and Wargo, 2015) in those treatments. A wide variety of BFT metabolites revealed BFT potency as a source for generated bioactive molecules (telmisartan, LCA) as they are functional molecules in aquatic biosecurity and metabolism and a reservoir for other bioactive molecules (bile acids) as they are functional molecules in lipid metabolism and aquatic biosecurity in BFT. Many of the identified BFT molecules have not have their relationship to aquatic bacterial activities and shrimp metabolism investigated in the literature. Based on the available literature, some of those molecules are discussed in this study.

Biofloc volume, which serves as an indicator of heterotrophic bacterial activity, fluctuates with temperature due to its influence on bioflocculation activity. Additionally, biofloc is partially consumed by shrimp, supplying bioactive molecules to their digestive systems (Hostins et al., 2015). Heterotrophic bacterial activities mainly depend on organic carbon availability. Starch was selected as a chemically defined, readily degradable carbon source with consistent quality, in contrast to more complex commercial sources such as molasses. This choice provided a clear benchmark for carbon availability against which the performance of seaweed, a structurally complex substrate with a slower carbon release profile and associated bioactive compounds, could be evaluated. The final body weight was significantly higher at 28 °C than at 22 °C temperature treatments. This increase was mainly due to lower shrimp density combined with the same amount of feed provided across both temperatures, along with the stimulatory effect of warmer conditions on feed intake, which in turn enhanced growth (Hostins et al., 2015; Prates et al., 2023). The survival rate surpassed 84% at 28 °C, aligning with findings from prior studies on *Penaeus vannamei* in biofloc technology (BFT) systems (Ponce-Palafox et al., 2019). In contrast, treatments at 22 °C exhibited even higher survival rates, reaching up to 92%. These results are consistent with a study investigating the impact of varying temperatures on the survival rate of *P. semisulcatus* (Kumlu et al., 2000). The current study recorded cannibalistic behavior in the 28 °C treatment group, which was not observed in the 22 °C group. However, lower survival at 28 °C could be additionally explained by the interaction of the biofloc system and shrimp immunity (Faramudhita et al., 2026). The higher temperature (28 °C) significantly enhanced SGR across all carbon source treatments. However, superior growth at 28 °C coincided with lower survival rates than the 22 °C groups. This is likely due to the interactive stress of higher metabolic demand at warm temperatures combined with the interaction of microbial community with shrimp immune system (Chen et al., 2021; Faramudhita et al., 2026). Furthermore, while dietary protein supported growth, the PER and FCR were optimal in the Starch-28 treatment, indicating that efficient nutrient utilization under favorable temperature conditions is the key driver for maximizing biomass yield.

### 4.2 BFT molecules profile

Recognized bioactive molecules in BFT showed a relationship with bacterial composition in shrimp intestine. In the BFT environment, these molecules could be partly consumed by the BFT bacterial community (Colombo et al., 2023). Shrimp can capture biofloc in BFT systems based on their body size (Krummenauer et al., 2020), contributing to the body biomass by 22%–43% as C, and 0%–43% as N at nursery-based *L. vannamei*, but 63%–100% as C, and 35%–86% as N in adult *L. vannamei* (Abakari et al., 2022). This would indicate a direct delivery of bioflocs to the intestinal tract in larvae and adult shrimps. The viable microbes and dead bacterial cells with their metabolic content could be regarded as important elements in the environment–host microbial interaction through ingestion by shrimp (Tepaamorndech et al., 2020). For example, BFT water which showed high *Vibrio* abundance also increased *Vibrio* abundance in Pacific white shrimp intestine (Tepaamorndech et al., 2020).

In the current study, starch-22 treatment exhibited an increase in lipid content compared to other treatments, with lauric acid being particularly abundant, serving as a key functional compound within the lipid category. This increase can be attributed to the positive impact of lower temperatures on the growth of ammonia-oxidizing bacteria, which coexist with algae, leading to elevated levels of lauric acid. Notably, lauric acid has been shown to reduce *Vibrio* abundance in the intestines of swimming crabs (Zhan et al., 2024). The optimal temperature for ammonia oxidizing bacteria is lower than that for heterotrophic bacteria. At 28 °C, heterotrophic bacterial growth restricts the ammonia oxidizing bacterial growth regarding competition for nitrogen nutrients. Notably, the dominance pf biofloc heterotrophic bacteria over ammonia oxidizing bacteria is not likely due to organic carbon toxicity (Soaudy et al., 2023). It is recommended that future studies perform BFT bacterial and algal composition analysis to reveal the relationship between the biofloc chemical bioactive molecules and biofloc system performance-based bacterial and algal activities.

A temperature of 28 °C boosts the activity of heterotrophic bacteria on seaweed material as a carbon source, promoting the release of additional beneficial molecules into the biofloc medium (Arandia-Gorostidi et al., 2020). Organic acids and their derivatives combined with organoheterocyclic compounds were highest level in Mix-28 treatment. Organic acids have several benefits for shrimp, including growth, nutrient utilization, and disease resistance (Ng and Koh, 2017). Organoheterocyclic compounds have antibacterial, antioxidant, and anti-inflammatory effects (An et al., 2024). Seaweed is a rich source of those chemical compounds with antioxidant properties of benzenoids (Kannan et al., 2024; Yaakob et al., 2014).

These combined effects may account for the enhanced growth observed in the Mix-28 treatment. Seaweed-28 showed the highest level of telmisartan in BFT and highest Firmicutes/Bacteroidota ratio in the intestine of shrimp, which could indicate a healthy microbial composition in their intestine. Telmisartan induces the activity of intestinal commensal microbiota, including the Firmicutes/Bacteroidota ratio in rats (Beckmann et al., 2021), and this bacterial ratio is higher in healthy individual piglets than in infected ones (Molist et al., 2012). Telmisartan blocks bacterial lipopolysaccharide receptor, which decreases the production of nitric oxide and interleukin-1β in murine macrophages (Choe et al., 2019). Nitric oxide is an important molecule in the metabolism of pathogenic bacteria, including *Vibrio cholerae* and *Pseudomonas aeruginosa* (Williams and Boon, 2018). Cadmium and arsenic pollution will affect telmisartan availability in relation to the chelating effect (Fouad et al., 2015; Fouad and Jresat, 2011). In aquaculture regions which are cadmium and arsenic polluted environments, telmisartan availability is expected to be an ineffective supplement, which should be considered in future studies.

The Mix-28 and Starch-22 treatments exhibited elevated levels of two bile acids alongside reduced *Vibrio* bacterial abundance. Hyocholic bile acid, a primary bile acid species in pigs, suggests the presence of commercial feeds supplemented with bile acids (Li et al., 2023; Zhang et al., 2023), a common feed additive in China. Recently, bile acids and salts have been introduced as innovative feed additives in the animal health industry, primarily functioning as emulsifiers that enhance lipid utilization. In the current study, the Mix-28 and Starch-22 treatments appeared to lack bile-acid-metabolizing bacteria, which helped preserve bile acids in the BFT medium and controlled the abundance of pathogenic bacteria. Bile acids may play a specific role in disease control, as they exhibit a dialysis effect on the cell walls of pathogenic bacteria (di Gregorio et al., 2021), a chelation reaction with virulence factors (Cahill, 1983; Glatman-Freedman et al., 2000; Kocsár et al., 1969; Van Bossuyt et al., 1988), a catabolizing reaction on virulence factors (Mukherji and Prabhune, 2015), and even an interaction with host immune systems that mediates the side effect of virulence factors on host immunity (Anisfeld et al., 2005; Nagarajan et al., 2022; Peng et al., 2019; Peric-Golia and Jones, 1962; Rudling et al., 2019; Zhu et al., 2019). Most of these results require investigation for practical applications in aquaculture as feed or water additives and their effects on BFT performance and biosecurity. In this study, Starch-22 and Mix-28 showed the highest level of two bile acids, including hyocholic acid and chenodeoxycholic acid. The elevated levels of these acids were associated with the lower *Vibrio* abundance in shrimp intestine in the related treatments, which could be explained by the lower bile acid catabolizing bacterial abundances in related treatments, with this bile acid availability in BFT facilitating control over *Vibrio* bacteria. Further research is required to investigate the effect of bile acid supplementation on pathogenic bacterial abundances in both shrimp intestine and culture water with the aim of improving the biosecurity level of shrimp farms. The phyla Bacteroidota and Firmicutes contain known bile acid-oxidizing bacteria (Hylemon et al., 2018; Solenne et al., 2020), but their overall abundance in treatments does not clearly indicate enriched oxidative function. For instance, Bacteroidota was lower in Starch-28 and Mix-22, while Firmicutes was lower in Starch-28, Seaweed-22, and Mix-28. Only Mix-28 showed a profile where Firmicutes was present at level that might support this activity. However, because these phyla include many species without this specific function, the observed taxonomic shifts do not confirm the presence or activity of bile acid oxidizers. Future studies should directly measure bile acid oxidation genes or metabolites in biofloc systems to draw definitive conclusions.

The elevated LCA levels observed in the Mix-28 treatment, compared to Mix-22, indicate a bacterial community under minimal stress with sufficient access to nitrogen and carbon substrates. The LCA likely originated from the bacterial metabolism of feed or fecal matter within the system. The available nitrogen and carbon sources could allow LCA to be protected from bacterial catabolic activity and increase the likelihood of its re-ingestion by shrimp feeding on BFT flocs. LCA can be utilized by both Gram-positive and -negative bacteria for various physiological functions under both anaerobic and aerobic conditions. In contrast, Mix-22 exhibited elevated levels of LCA catabolic by-products. LCA can be metabolized as a carbon or nitrogen source, or even as an electron acceptor (Flanagan et al., 2010). Some bacteria utilize LCA as a nitrogen source by metabolizing LCA to glycine, which is catabolized to serine and then to the pyruvate and ammonia molecules (Meadows and Wargo, 2015). Other bacteria catabolize the LCA molecule as a carbon source, such as Betaproteobacteria, Gamma-proteobacteria, and Firmicutes (Zhu et al., 2014). This metabolic pathway is subjected to one of two mechanisms. First, LCA is metabolized to the trimethylamine and malic semialdehyde through the bond cleavage between the nitrogen and carbon atoms (Meadows and Wargo, 2015). Second, LCA is metabolized to a glycine betaine by a carnitine dehydrogenase enzyme in the presence of ATP or CoA molecules (Lindstedt et al., 1967; Meadows and Wargo, 2015). In the non-appearance of these two molecules, LCA is metabolized to trimethylaminoacetone and CO_2_ through decarboxylation (Lindstedt et al., 1967). Among LCA catabolic byproducts, trimethylamine (LCA as a carbon source) and pyruvate (LCA as a nitrogen source) showed higher levels in Mix-22 than in Mix-28 treatment. Additionally, gammaproteobacterial abundance was higher at Mix-22 than at Mix-28—these are intestinal microbiota that adapt to stress by importing or synthesizing a compatible solute such as LCA (Brown and Simpson, 1972); bacteria use LCA as a compatible solute (Warren, 2013a; 2013b). At low temperatures, carnitine forms (D-and L-) can be imported through the OpuC transporter, which is classified as an ATP-binding cassette transporter family member (Angelidis and Smith, 2003; Kappes and Bremer, 1998). LCA basically functions in lipid metabolism by transferring long-chain fatty acids to the mitochondrial matrix where β-oxidation takes place. LCA protection against negative bacterial effects requires a moderate quantity of dietary fiber with the aim of sparing it for shrimp growth instead of consumption by gut bacteria, since low dietary fiber will decrease the carbon source availability for gut bacteria and present the LCA molecules as an alternative carbon source. Additionally, the high dietary content of fiber will increase gut bacterial growth to the extent of producing a bacterial molecule to compete with LCA in its transporter (organic cation transporter novel 2, OCTN2) capacity (Ghonimy et al., 2018). Additionally, dietary free minerals represent a chelating interaction with LCA molecules, affecting their absorption and metabolism at gut, tissue, and mitochondrial levels (Tjale et al., 2022). A new practice in LCA protection is required during diet manufacturing to decrease LCA loss from feeds or in feces.

Mix-28 showed an increased level of mesaconic acid compared to Mix-22. Mesaconic acid is a bacterial substrate, which *Methylorubrum extorquens*, pink-pigment bacteria, produce carotenoids (Mo et al., 2020). These bacteria produce mesaconic acid and re-uptake it during exponential growth phase (Pöschel et al., 2022). This might indicate a doubling of the bacterial population and generate extra carotenoids, which are essential molecules for crustaceans, not only as pigment that has marketing value but also as a growth promoter and antioxidant agent (Ghonimy et al., 2023b). Astaxanthin-producing bacteria are easily exposed to osmotic stress at a salinity level of 17 ppt (Bruger et al., 2024). However, the current study revealed that under high salinity conditions, the level of mesaconic acid—an indicator of astaxanthin-producing bacteria—increased. This suggests that adding Mix-28 effectively supported the natural production of carotenoids.

Mix-22 showed the highest level of isobutyric acid, along with a high abundance of *Vibrio* species compared to other 22 °C treatments. The a-aminoisobutyric acid is a bacterial product which is transported within the bacterial cell of the marine bacterium *Vibrio alginolyticus* (Guo et al., 2011; Tokuda et al., 1982). The use of complex carbon sources under low-temperature conditions should be avoided, as their slow release rate can limit heterotrophic bacterial growth and reduce their competitiveness against *Vibrio*.

### 4.3 Microbiota composition and bacterial diversity in shrimp intestine

The carbon source and temperature in BFT improve and maintain a stable and beneficial microbiome composition by regulating the production of specific bioactive compounds that directly and indirectly influence bacterial communities. For example, the carbon source determines the availability of key molecules: when using a mix (starch–seaweed) carbon source at 28 °C compared to starch at 28 °C, the compound L-carnitine (fold-change: 30.0) is significantly upregulated. Its biological effect is as a versatile carbon source, nitrogen source, and electron acceptor, supporting a wide range of beneficial bacteria such as *Pseudomonas* sp., *Lactobacillus plantarum*, and *Rhizobium* sp., thereby promoting a diverse and functional community. Similarly, 1,4-D-xylobiose (fold-change: 51.0 in Mix-28 vs. Starch-28) is a complex sugar that serves as a carbon source for beneficial Bifidobacteria and *Lactobacillus*. The temperature interacts with the carbon source to amplify these effects; for instance, comparing Mix-28 to Mix-22, L-carnitine shows an extremely high fold-change (450), indicating that elevated temperatures dramatically increase the production of this multi-functional nutrient and thus further stimulate beneficial bacterial metabolism. Furthermore, certain carbon sources help suppress harmful elements: in Seaweed-28 vs. Starch-28, the antioxidant didodecyl 3,3′-thiodipropionate oxide (Fold-change: 12.0) has a general antioxidant effect that protects bacterial metabolic systems from stress, thus indirectly supporting community stability. Conversely, the profile of inhibitors is also shaped; for example, in Starch-28 vs. Starch-22, the antibiotic by-product desethylene ciprofloxacin is downregulated (Fold-change: 11), reducing a potential inhibitory pressure on the microbiome. Thus, by selectively promoting beneficial activators (such as carbon/nitrogen sources) and reducing inhibitors (such as antibiotic residues), the interplay of carbon source and temperature tailors the molecular environment to foster a stable and beneficial microbiome.

Heterotrophic bacteria produce poly-β-hydroxybutyric acid (PHB), which inhibits *Vibrio* infection (Avnimelech, 2015; Halet et al., 2007). In our study, Mix-28 showed the highest proteobacterial abundance (many of these bacteria are heterotrophic bacteria) and a higher 2-(2′,3′,4′-trihydroxybutyl) quinoxaline (quinoxaline derivatives with antibacterial activities) level with lower *Vibrio* abundance than Seaweed-28. Mix-22, however, showed a higher level of 2-(2′,3′,4′-trihydroxybutyl) quinoxaline with lower *Vibrio* abundance compared to Seaweed-22, but Mix-22 did not show a higher proteobacterial abundance than Seaweed-22. This lower proteobacterial abundance could be explained by the greater competitive activity of *Vibrio* species relative to general heterotrophic bacteria under low-temperature condition. Basically, heterotrophic Proteobacteria and Bacteroidota compete with bacterial pathogens for nutrients and space (Emerenciano et al., 2013; Wei et al., 2016), and these factors affect heterotrophic growth and, subsequently, *Vibrio* abundance (Panigrahi et al., 2020).

In this study, numerous BFT metabolites, including xenobiotics, are linked to bacterial activities in the literature. However, the relationship between these metabolites and bacterial activities in both aquatic environments and animal digestive tracts remains largely unexplored. Future research is needed to uncover the impact of BFT-derived metabolites on bacterial activities in aquaculture systems and their influence on the immune status of farmed species, as these molecules hold potential as prebiotics in aquatic diets. Additionally, the bioremediation capabilities of biofloc systems are highly promising, as this closed environment can create a fully sustainable and eco-friendly setting through bacterial activity. Further studies are essential to examine the effects of various nutritional and environmental factors on the bioremediation potential of biofloc systems, thus contributing to advances in the green economy and food safety. Despite the biosecurity merit in BFT, a few pathogenic infestations and disease outbreaks cases in BFT have been reported (Aguilera-Rivera et al., 2019; Hoang et al., 2020). *Vibrio harveyi*, *V. rotiferianus*, *V. alginoticus*, *Photobacterium* sp., and *Photobacterium damselae* have been reported in the commercial production of *L. vannamei* BFT (Aguilera-Rivera et al., 2019; Ferreira et al., 2015). In this context, investigating the interaction mechanism between culture water and shrimp intestinal microbiota composition is important issue; chemical and nutrient availabilities in BFT could help reveal the pathogenicity mechanism in cultured shrimp. In this study, the phenolic compounds of biofloc showed a higher level in Mix-28 than other 28 °C treatments, with a lower *Vibrio* abundance than all treatments, whereas Seaweed-22 showed a higher level with different phenolic compounds than other 22 °C treatments. Since most brown algae exhibit a high phenolic content with an antimicrobial activity (Hostins et al., 2015), further investigation is required to reveal the effectiveness of different phenolic compounds on bacterial activity, especially benzenoids, 2-propylphenol, and methoxyphenol.

This study detected numerous bioactive compounds with diverse biological activities. Since the feed and water source were identical for all experimental groups, these xenobiotics represent a controlled background input into the system. Based on untargeted molecule analysis, Seaweed-22 showed the lowest bioremediation effect on the pesticide (imazethapyr) and an elevated level of an antioxidant (thiodipropionate), while Seaweed-28 showed an elevated level of antibacterial agent (nitrophenol), and Mix-22 showed an elevated level of an antibacterial agent (fosetyl), metabolic enhancer (isobutyric acid), and beneficial bacterial enhancers (melibiose, piperidinecarboxamide), in addition to a low bioremediation effect on sulfamethazine antibiotic. Mix-28 showed a typically high level of LCA as lipid metabolism enhancer and elevated levels of beneficial bacterial enhancer (xylobiose) and antibacterial agents (irisflorentin, galactinol, xylobiose, and propranolol), in addition to a high bioremediation effect on methylisoxazol (byproduct of drug oxidation) and lower bioremediation effect on cholesteryl sulfate (antifungal). It is recommended that future studies address BFT bioremediation capacity and the safety level of each contaminant in avoiding their negative effects on shrimp growth and food safety for human consumption. As BFT is a closed environment, it accumulates different molecules during the whole culturing period. Considering the circular economy and green aquaculture practice concepts, the accumulated molecules could be ingested by shrimp for further metabolic and intestinal microbiota benefits or be treated by BFT bacteria for bioremediation benefits.

Bacterial diversity was assessed using the Shannon index and species richness indicators. In this study, richness indices (Chao 1, Simpson) were generally higher in the 28 °C treatments except for Seaweed-28. This suggests that mixed carbon sources (with their varying complexities and availabilities) support greater microbial diversity in biofloc systems. In contrast, the Shannon index was lower in Seaweed-28 and Mix-28, reflecting the influence of seaweed as a slowly degraded carbon source on shaping the effective number of bacterial groups within the community. Notably, Mix-28 also exhibited the lowest *Vibrio* abundance in shrimp intestine among all 28 °C treatments, underscoring the importance of sustained carbon release from diverse and complex carbon sources in modulating bacterial community structure and potentially suppressing pathogenic *Vibrio*.

The high proteobacterial abundance was associated with the higher biofloc volumes in the related 28 °C treatments, indicating activated heterotrophic bacterial activities. Proteobacteria are dominant bacteria in crustaceans. It has been reported that Alphaproteobacteria are the dominant bacteria in healthy *L. vannamei* shrimp, while Gammaproteobacteria are dominant in *L. vannamei* and *Penaeus monodon* shrimp intestine (Li et al., 2024), while infected shrimp show dominant phyla of Firmicutes and Bacteroidota (Hossain et al., 2021).

The high proteobacterial abundance in biofloc systems, as discussed previously, underscores the importance of a balanced gut microbiome for shrimp health. The interaction between shrimp intestinal microbiota and immune response is crucial for health and disease resistance. Probiotic supplementation in biofloc enhances immune parameters by modulating the gut microbial community. This beneficial shift in microbiota reduces pathogenic *Vibrio* abundance and increases the richness of beneficial bacteria, which in turn strengthens the shrimp’s non-specific immune defenses (Faramudhita et al., 2026). Since the current study showed limitations in directly measuring these immune parameters, we recommend that future studies incorporate comprehensive immune assays. This approach will not only bridge the gap between observed microbial shifts and host health outcomes but also provide a mechanistic link to the following discussion on novel disease resistance strategies, such as the chelation of bacterial virulence factors.

This study specifically conducted its examinations at 22 °C and 28 °C because these temperatures represent critical operational thresholds in commercial Chinese biofloc shrimp farming, where issues with system performance and *Vibrio* outbreaks are frequently reported. By focusing on these temperatures, we directly addressed these practical concerns. For future research, we recommend including an intermediate temperature (e.g., 25 °C) to refine the thermal response curve and provide deeper insight into how temperature precisely modulates biofloc biosecurity efficacy.

In the current study, treatments with lower *Vibrio* abundances were accompanied by a higher proteobacterial abundance in Mix-28 and Starch-22 treatments. In addition, BFT showed variety in its bioactive molecule composition related to bacterial cell activity or bacterial virulence factors; some of these bioactive molecules were associated with a lower *Vibrio* abundance. For example, irisflorentin and probucol are anti-agents against the bacterial virulence factor of LPS, associated with a lower *Vibrio* abundance in the Mix-28 treatment. In humans, manipulating the availability of the virulence factor (pyoverdine) feeds back on a decreased pathogenic bacterial load, but with a high level of elimination, the host immune system further overreacted since a minimum level of virulence factor is required for immune strength (Weigert et al., 2017). In this context, a new concept of disease resistance could be raised in applications of aquaculture disease control, and so the chelating of virulence factors could be a new strategy in disease control.

Based on our results, the synergistic effects of the identified bioactive compounds likely contribute to improved shrimp weight gain and reduced intestinal *Vibrio* abundance. For optimal carbon management in BFT, a temperature-dependent strategy is recommended. During the low-temperature system preparation phase, simple carbon sources such as molasses should be used to rapidly boost heterotrophic bacterial growth and enhance their competitiveness against pathogens such as *Vibrio*. Once a stable cultivation temperature of 28 °C is reached, a blended approach utilizing both simple and complex carbon sources is advised. This combination ensures a sustained release of carbon, which helps maintain a robust and stable heterotrophic bacterial community. A resilient heterotrophic community is more effective at outcompeting pathogenic bacteria for resources and can better synergize with dietary bioactive compounds (based on current study nominated bioactive molecules for *Vibrio* control) to consistently suppress *Vibrio* growth throughout the production cycle. This integrated approach, combining strategic carbon source management with bioactive additives, creates a favorable microbial environment that supports shrimp health, growth, and sustainable production in BFT systems. For enhanced biosecurity, a novel strategy involves using feed or water additives, which are designed to chelate or neutralize bacterial virulence factors (such as bile acids, irisflorentin, and probucol) to control disease.

## 5 Conclusion

Temperature and carbon source strongly influenced bioactive molecule profiles and intestinal microbiota in *Penaeus vannamei* BFT culture. Starch-22 and Mix-28 effectively reduce *Vibrio* and enhance beneficial bacteria under low and high temperatures, respectively. Key bioactive molecules, including mesaconic acid, irisflorentin, probucol, telmisartan, and bile acids, correlate with improved shrimp health and disease resistance. These compounds show great potential as prebiotics for promoting microbial balance and suppressing bacterial virulence. Strategic carbon management, simple carbon during the low-temperature BFT phase (onset of culture season), and mixed carbon at 28 °C during BFT running phase sustain a stable heterotrophic community. Virulence factor chelation by additives such as bile acids, irisflorentin, and probucol offers a novel biosecurity approach. We recommend adopting temperature-adapted carbon regimes and bioactive supplementation in commercial BFT systems.

## Data Availability

The original contributions presented in the study are publicly available. This data can be found here: https://www.ncbi.nlm.nih.gov/sra/PRJNA1456873.
